# Targeting of REST with rationally-designed small molecule compounds exhibits synergetic therapeutic potential in human glioblastoma cells

**DOI:** 10.1186/s12915-024-01879-0

**Published:** 2024-04-12

**Authors:** Svetlana B. Panina, Joshua V. Schweer, Qian Zhang, Gaurav Raina, Haley A. Hardtke, Seungjin Kim, Wanjie Yang, Dionicio Siegel, Y. Jessie Zhang

**Affiliations:** 1 Department of Molecular Biosciences, The University of Texas at Austin, 2500 Speedway, Austin, TX USA; 2 Skaggs School of Pharmacy and Pharmaceutical Sciences, The University of California San Diego, 9500 Gilman Drive 0741, La Jolla, CA USA

**Keywords:** Glioblastoma (GBM), Repressor element-1 silencing transcription factor, SCP1 phosphatase, Synergy

## Abstract

**Background:**

Glioblastoma (GBM) is an aggressive brain cancer associated with poor prognosis, intrinsic heterogeneity, plasticity, and therapy resistance. In some GBMs, cell proliferation is fueled by a transcriptional regulator, repressor element-1 silencing transcription factor (REST).

**Results:**

Using CRISPR/Cas9, we identified GBM cell lines dependent on REST activity. We developed new small molecule inhibitory compounds targeting small C-terminal domain phosphatase 1 (SCP1) to reduce REST protein level and transcriptional activity in glioblastoma cells. Top leads of the series like GR-28 exhibit potent cytotoxicity, reduce REST protein level, and suppress its transcriptional activity. Upon the loss of REST protein, GBM cells can potentially compensate by rewiring fatty acid metabolism, enabling continued proliferation. Combining REST inhibition with the blockade of this compensatory adaptation using long-chain acyl-CoA synthetase inhibitor Triacsin C demonstrated substantial synergetic potential without inducing hepatotoxicity.

**Conclusions:**

Our results highlight the efficacy and selectivity of targeting REST alone or in combination as a therapeutic strategy to combat high-REST GBM.

**Supplementary Information:**

The online version contains supplementary material available at 10.1186/s12915-024-01879-0.

## Background

Glioblastoma (GBM) is the most common primary malignant brain tumor in adults, with an incidence rate of 3.7 per 100,000 person-years and a high mortality rate [1]. GBM exhibits high resistance to conventional radiation and chemotherapy. Emerging evidence suggests that metabolic reprogramming or adaptation may contribute to therapy resistance in GBM [2]. Additionally, GBM is characterized by great intratumoral molecular and metabolic heterogeneity, further contributing to its high lethality [2]. Given these facts, novel therapeutic approaches targeting deregulated cellular pathways must be explored to improve patient prognosis and eventually develop treatments for this fatal disease [2, 3].

One of the deregulated genes in GBM is a repressor element-1 silencing transcription factor (REST), a transcriptional repressor that has been identified as an oncogenic protein in various brain tumor types, including neuroblastoma, medulloblastoma, and glioblastoma [4, 5]. High expression of REST was significantly associated with worse overall survival, progression-free interval, and worse disease-specific survival in glioma patients [6]. Targeting REST may inhibit cancer stem cell proliferation as REST is crucial for cancer stem cell self-renewal [7]. Chronologically, one of the initial studies on REST in glioblastoma stem cells (GSC) demonstrated that GSC with high REST expression produced more invasive tumors compared to those with low REST expression in orthotopic mouse tumor models [8]. Genetic knockdown of REST in high-REST GSCs resulted in increased survival of mice [8]. Importantly, treatments targeting REST may have less severe neurological side effects than conventional chemotherapy because post-mitotic neurons do not express REST [7]. Therefore, reducing REST levels in high-REST glioblastoma tumors holds promising therapeutic effects.

The REST protein acts as a transcription factor, silencing the neuronal gene expression [9]. Unlike enzymes, targeting transcription factors with small molecule inhibitors has historically been challenging [10]. However, prior studies have shown that REST level is post-translationally regulated by phosphorylation-dependent protein turnover [11, 12]. Once phosphorylated, REST is targeted to the cytosol for degradation by the ubiquitin ligase SCF^*β*−TrCP^ [11, 12]. Thus, the chemical modulation of the REST protein level can be achieved by regulating its phosphorylation. One potential molecular approach to reduce REST involves targeting C-terminal domain small phosphatase 1 (CTDSP1/SCP1), which dephosphorylates REST at sites, such as Ser861 and Ser864, that function as checkpoints for REST degradation [12, 13]. REST lacking phosphorylation at Ser-861 of Ser-864 becomes more stable [13], and REST stabilization can be prevented by inhibiting SCP1’s phosphatase activity, leading to a reduced REST protein level. To this end, we have previously designed an initial series of compounds called the T-series of small molecule covalent inhibitors of SCP1 [14]. These compounds demonstrated the capability to inhibit SCP1 phosphatase activity and decrease REST protein levels in human HEK293 cells.

In this study, we started with a well-characterized high-REST glioblastoma cell line (T98G) to validate the role of REST in glioblastoma growth. We used CRISPR/Cas9 gene editing to generate homozygous REST-null single-cell clonal lines from T98G and non-neural HEK293 cells and compared their transcriptomes. We demonstrated that REST knockout significantly impaired the proliferation of GBM cells. We developed a new optimized chemical lead (GR-28) that causes degradation of REST protein in REST-dependent glioblastoma cells via covalent inhibition of SCP1. The GR-28 compound degraded cellular REST protein, derepressed REST-silenced genes, and induced cell death in high-REST GBM cells. We also showed that some REST-null clones were able to rewire fatty acid metabolism to derepress their growth and that this compensation effect could be negated using a chemical inhibitor of long-chain acyl-CoA synthetases, Triacsin C. GR-28 exhibited profound synergy when combined with Triacsin C in GBM cells, but not in hepatocarcinoma cells (HepG2), allowing effective eradication of glioblastoma cells with limited hepatotoxicity in vitro.

## Results

### REST is upregulated in TCGA-LGG/GBM samples and select glioblastoma cell lines

**Fig. 1:**
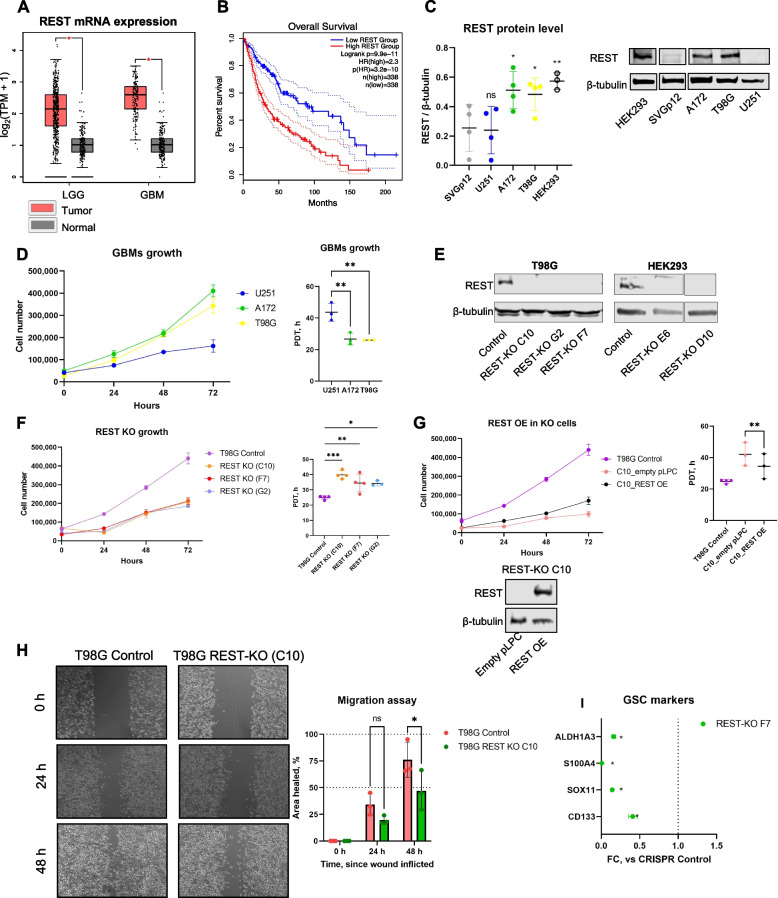
REST promotes glioblastoma growth. **A** Boxplot of REST mRNA expression in TCGA-LGG and TCGA-GBM samples compared to normal brain samples from TCGA and GTEx datasets (*p < 0.001). **B** Survival analysis using data from TCGA-GBM and TCGA-LGG projects. Analysis was done using GEPIA2 web server (**A**,** B**). **C** Basal REST protein amount in a panel of select cell lines. Three to four independent biological replicates are shown as mean ± SD. Statistical difference vs SVGp12 was tested using ANOVA with post hoc tests. Individual data values are provided in Additional File 10A. **D** Proliferation of GBM cell lines assessed by counting cells every 24 h. Shown is one representative replicate and quantification of PDT based on three independent experiments (mean ± SD). Statistical difference vs U251 was tested using ANOVA with post hoc tests. Individual data values are provided in Additional File 10B. **E** Western blot confirms the lack of REST in homozygous REST-KO clones of T98G (*left*) and HEK293 (*right*). **F** Proliferation of WT and REST-KO T98G cells was examined by counting cells every 24 h. Shown is one representative replicate and quantification of PDT based on three to four independent experiments (mean ± SD). Statistical comparison vs T98G control was performed using ANOVA with post hoc tests. Individual data values are provided in Additional File 10C. **G** Proliferation of T98G WT and REST-KO C10 cells (transfected with empty vector pLPC vs REST OE) was examined by counting cells every 24 h. Shown is one representative replicate and quantification of PDT based on three independent experiments. Statistical difference was tested using two-tailed paired *t*-test. Individual data values are provided in Additional File 10D. **H** Wound scratch assay and its quantification using ImageJ. Shown are mean ± SD from three independent biological experiments. Groups were compared using paired *t*-tests. Individual data values are provided in Additional File 10E. **I** Effect of REST loss on GSC marker expression. Shown are fold changes (FC) vs CRISPR Control derived from three independent biological replicates. Comparison vs control was performed using unpaired one-tailed *t*-tests. Dashed line indicates FC = 1. Individual data values are provided in Additional File 10F. ***p < 0.001; **p < 0.01; *p < 0.05; ns—not significant

To analyze the impact of REST on GBM growth, we conducted a bioinformatics analysis of The Cancer Genome Atlas database (TCGA-GBM and TCGA-LGG projects) and compared REST mRNA abundance in low-grade glioma/LGG samples (*n* = 518) and GBM samples (*n* = 163) against normal brain samples (*n* = 207) from two combined datasets—TCGA and GTEx (Genotype-Tissue Expression project [15]). We observed significantly elevated REST gene expression in both low- and high-grade glioma (*p* < 0.001), Fig. 1A. Consistent with other published analyses [6], higher REST expression was associated with worse overall survival in the pooled population of patients with low-grade glioma (LGG) and glioblastoma (logrank *p* = 9.9e − 11, Fig. 1B). Survival analysis for separate LGG and GBM subsets is shown in Additional File 1: Fig. S1. The lack of REST association with GBM patients survival is likely explained by threefold lower sample size of TCGA-GBM subset vs TCGA-LGG.

REST is a silencing transcription factor that suppresses the expression of neuronal genes, and its expression is ubiquitous in non-neural tissues but down-regulated in neural precursors and neurons [16]. We measured REST protein amount in several well-characterized GBM cell lines compared to control cell lines—non-neural HEK293 cells and glial SVGp12 cells. Three GBM cell lines were included in the study: U251, A172, and T98G. Two of the cell lines—A172 and T98G—had significantly upregulated basal REST protein amount compared to SVGp12 cells (*p* < 0.05) (Fig. 1C). The REST expression level was also high in HEK293 cells since REST is ubiquitously expressed in non-neural cells. Interestingly, proliferation assays showed that the two high-REST GBM cell lines (A172 and T98G) proliferate faster than low-REST U251 cells (Fig. 1D). Taken together, these results suggest glioblastoma cells vary in their REST protein level.

### REST promotes GBM cell proliferation in vitro

To study the effect of REST on GBM proliferation, we used CRISPR-Cas9 to generate REST-knockout (REST-KO) homozygous cells from a representative glioblastoma cell line, T98G, containing high REST protein amount. To identify the genes specific to GBM rather than common REST targets, we also generated the REST-KO for non-neural HEK293 cells, which also contain a high REST amount (Fig. 1E). Accordingly, two single-guide RNA (sgRNA) oligonucleotides targeting two specific human REST genomic regions located in exons 2 (sgRNA RY1) and 3 (sgRNA RG6) were synthesized and cloned into pX330 vector [17]. Target cells (T98G or HEK293) were either (1) co-transfected with Cas9-2A-GFP and empty pX330 vector (CRISPR control) or (2) co-transfected with Cas9-2A-GFP and two sgRNA expression vectors for double-nicking recombination (REST-KO). Western blotting confirmed the absence of the protein band corresponding to the observed REST molecular weight, ~ 200 kDa (Fig. 1E, Additional file 1: Fig. S2A-B). To identify the DNA sequences of selected REST-KO clones, we designed PCR primers flanking regions of sgRNA-introduced double-stranded breaks and amplified genomic DNA isolated from control or KO cells (Additional file 1: Fig. S2C). The resulting PCR products were purified and sequenced. In each case, the REST genomic sequence was repaired so that the resulting protein sequence had a premature stop codon (Additional File 2: Table S1**)**.

To evaluate the function of REST in GBM, we compared the cell proliferation rate in T98G WT and three different REST-KO clones, which showed that REST deficiency resulted in significant cell growth arrest, consistent with higher proliferation doubling time (PDT) (Fig. 1F**)**. To exclude potential off-target effects of CRISPR-mediated genome editing and further verify the specific effect of REST on promoting GBM proliferation, we reconstituted REST expression in REST knockout cells by transiently transfecting REST (Fig. 1G, *lower)*. We observed that the reconstitution of REST partly restored the cell proliferation rate in the REST-KO C10 clone (Fig. 1G**)**. The mean PDT of control cells was 24.9 h, whereas REST-KO C10 cells (transfected with empty pLPC) divided on average 1.7-fold slower. Furthermore, the reconstitution of REST rescued proliferation by approximately 56% (Fig. 1G). These observations suggest that REST is vital for GBM cell proliferation.

In addition, it has been reported that REST plays a significant role in migration and self-renewal of high-REST GBM cells [18, 19]. To further investigate the function of REST in GBM, we performed wound scratch assay and measured gene expression of commonly used GSC (glioblastoma stem cells) markers, respectively [20–23] (Fig. 1H–I). Our results corroborated published reports: REST loss led to slower migration of glioblastoma cells, as was estimated by wound scratch assay (Fig. 1H). Network analysis of genes commonly depleted in REST-KO clones showed that one of the most significant networks (PPI enrichment *p*-value: 3.09e − 11) was associated with cell migration (Additional File 1: Fig. S3A). Notably, *PDGFRA* and *FGFR1*, growth factor receptors tightly involved in GBM pathogenesis and therapy resistance [24, 25], were included in the network (Additional File 1: Fig. S3). Furthermore, gene expression of four tested GSC markers (*CD133, SOX11, ALDH1A3, S100A4*) was markedly reduced upon REST loss compared with wild-type glioblastoma cells (Fig. 1I).

Taken together, these data show that REST inhibition significantly hinders proliferation, migration, and stemness potential of GBM cells. Thus, targeting REST can have beneficial therapeutic effects in high-REST glioblastoma.

### Novel covalent inhibition of SCP1 with small molecule compounds

**Fig. 2:**
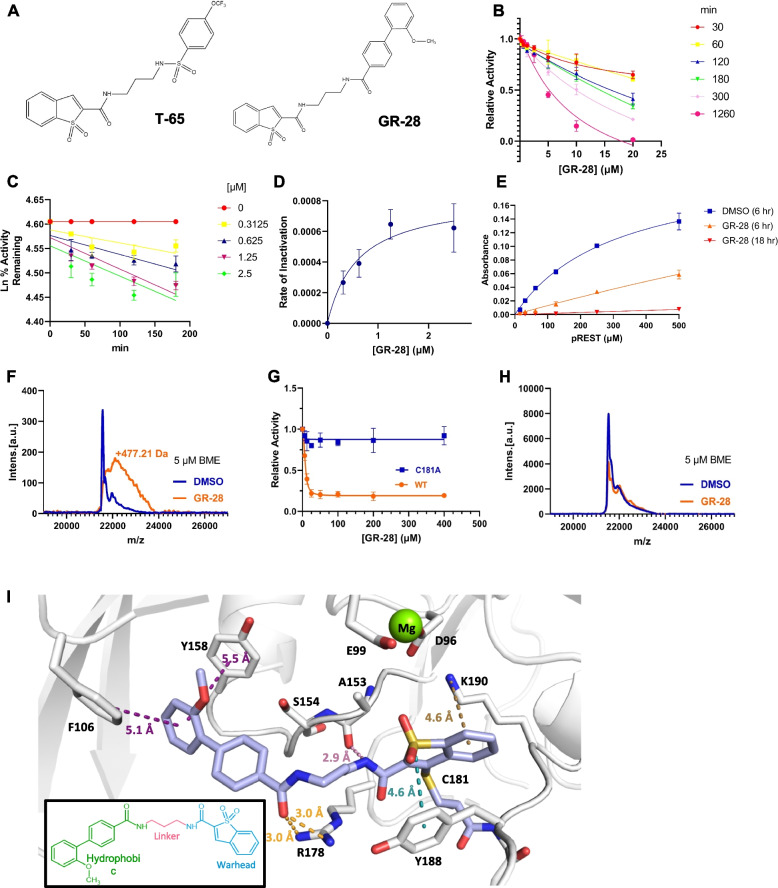
Novel covalent inhibition of SCP1 with small molecule compounds.** A** Chemical structures of lead SCP1 inhibitors. **B–D** Kinetic characterization of GR-28 against the phosphatase activity of SCP1. **B** Competitive assay against the *pNPP* substrate was performed with preincubation of GR-28 compound and SCP1 enzyme for 30, 60, 120, 180, 300, and 1260 min. Time-lapse IC_50_ curves were obtained. **C** The IC_50_ data of the *pNPP* substrate was converted to ln % remaining activity against time at different concentrations of GR-28 (0, 0.3125, 0.625, 1.25, and 2.5 μM). **D** The rate of inactivation (*k*_obs_) was plotted against inhibitor concentration and fitted to the equation: *k*_obs_ = *k*_inact_ × [*I*]/(*K*_*I*_ + [I]). Each data point was obtained from three replicates, and the error bars indicate SD. **E** The rate of inorganic phosphate generation measured by malachite green assay: SCP1 was pre-incubated with DMSO/GR-28 (20 μM), then samples were incubated with phosphorylated p-Ser861-REST. Each data point was obtained from three replicates, and the error bars indicate SD. **F** SCP1 WT was incubated overnight with GR-28 compound and then analyzed by MALDI-TOF MS. The blue trace represents the DMSO control, while the orange trace represents the GR-28 treatment. **G** *pNPP* assay of phosphatase activity of SCP1 WT (orange) or SCP1 C181A mutant (blue) upon incubation with GR-28 for 5 h. Error bars indicate SD from three replicates. **H** SCP1 C181A was incubated overnight with GR-28 compound and then analyzed by MALDI-TOF MS. **I** Model of covalent inhibition of SCP1 by GR28 (PDB Code: 3PGL). A magnesium ion is shown in the active site of SCP1 as a green sphere. Key residues predicted to interact with GR28 are shown as sticks. GR-28 is shown as sticks colored by atoms with carbon atoms in violet, oxygen in red, and nitrogen in blue. Side chain and main chain interactions between SCP1 and GR28 are shown in dashed lines

Though REST can be a therapeutic target for high-REST GBM due to its effect on proliferation, targeting transcription factors like REST by small molecules is often difficult. However, prior studies have shown that the cellular REST level is primarily regulated by its phosphorylation-triggered degradation. We rationally designed a series of small molecule covalent inhibitors (T-series) against SCP1 that were capable of reducing REST protein levels in human HEK293 cells [14] (T-65, Fig. 2A, *left*). These compounds have a hydrophobic moiety that recognizes the active site of SCP1 and places the warhead near a cysteine close to the active site allowing for covalent targeting. Despite our initial design efforts, T-65 exhibited limited cytotoxicity towards high-REST GBM cells (T98G and A172), and REST protein amount was not significantly changed after T-65 (4 µM, 24 h) treatment (Additional File 1: Fig. S4A-D**)**.

Starting from T-65, we synthesized (synthetic scheme in the Methods section) the second-generation focused library of ~ 20 compounds to delineate the structure–activity relationship (SAR). The scaffold was composed of three sections: a hydrophilic thiophene group as a warhead, a center linker comprised of a piperazine group, and a hydrophobic section comprised of an aromatic ring with various functional groups or halogen additions (Additional File 1: Fig. S4A). During the SAR analysis, we discovered that a hydrophobic aromatic ring was required for the potency of the inhibitors (group 1). Furthermore, inhibition strength was affected by the linker length in group 3. In our second-generation library, we varied the hydrophobic moiety of the compound to enhance its specific interaction with the SCP1 protein (Fig. 2A, *right*). When designing covalent inhibitors for SCP1, we aimed to promote the noncovalent interaction of designed compounds with SCP1 to first form a stable complex and second allow the slow formation of a covalent bond. Such design reduces the non-specific inhibition of SCP1 by only allowing covalent bond formation within the stably bound SCP1 complex.

To characterize the inhibition of SCP1 phosphatase by our new focused library of covalent inhibitors, we conducted para-nitro phenyl phosphate (*pNPP*) assays in a time- and concentration-dependent manner (Fig. 2B–D). We incubated the compounds with *pNPP* for different durations and then quantified the inhibition. Out of the focused library, compound GR-28 (Fig. 2A, *right*) exhibited the most inhibition and was characterized with *k*_inact_/*K*_*I*_ 1383 min^−1^ M^−1^ (Fig. 2B–D). To determine whether GR-28 primarily works on SCP1 to control REST phosphorylation, we employed malachite green assay, which measures the amount of inorganic phosphate released from phosphorylated REST peptide (pSer861-REST). As expected, the pre-incubation of SCP1 with GR-28 (20 µM) led to profound decrease in absorbance values, which was time-dependent (Fig. 2E). Therefore, we can conclude that GR-28 inhibits SCP1 capacity to dephosphorylate REST leading to accumulation of phospho-REST and its subsequent proteasomal degradation in vitro [13].

To test if the mechanism of SCP1 inhibition is a result of covalent bond formation [14], we incubated SCP1 with the inhibitor and measured the change in its molecular weight using matrix-assisted laser desorption ionization–time-of-flight mass spectrometry (MALDI-TOF MS). The prolonged incubation of GR-28 (theoretical molecular weight of 476.54 Da) with SCP1 led to a peak shift of 477.21 Da of mass. This shift was consistent with the formation of a covalent bond between the protein and the compound (Fig. 2F). Our previous studies have identified C181 nearby the active site as the target for covalent bond formation [14]. This was supported by our observation that the SCP1 C181A mutant shows resistance to GR-28 inhibition (Fig. 2G**)**. Furthermore, we conducted additional experiments to identify whether this resistance of the SCP1 C181A variant was attributed to the absence of adduct formation. Despite a prolonged overnight incubation of the SCP1 C181A variant with GR-28, no discernible covalent adduct peak was observed when analyzed with MALDI-TOF (Fig. 2H).

We further used molecular docking to model GR-28 inhibition of SCP1 (Fig. 2I). The X-ray crystal structure of SCP1 with its selective inhibitor rabeprazole provided us with a good initial template of the inhibitor binding pocket. GR-28 consists of a hydrophobic moiety, a linker, and a warhead (Fig. 2I, schematic). The end phenol ring of the hydrophobic moiety is located in a proximal pocket close to the active site, hydrophobically sandwiched between Y158 and F106. The middle ring of the hydrophobic moiety of the inhibitor does not provide direct interaction with the protein, but its rigidity reduces the entropy cost for compound binding. The amide bond of the hydrophobic moiety is stabilized by extensive interaction with the R178 side chain. The carbon linker is flexible, while the warhead moiety is well anchored to SCP1 when it covalently attaches to C181. The warhead forms a cation–π interaction with the benzothiophene ring and a π–π stacking interaction on the other side with Y188. The model also places the amide group of the warhead close to the carbonyl backbone of A153 with a potential hydrogen bond. The model suggests a strong interaction network upon the inhibitor covalently binding to SCP1.

### Transcriptome sequencing reveals distinct gene signatures associated with REST

**Fig. 3:**
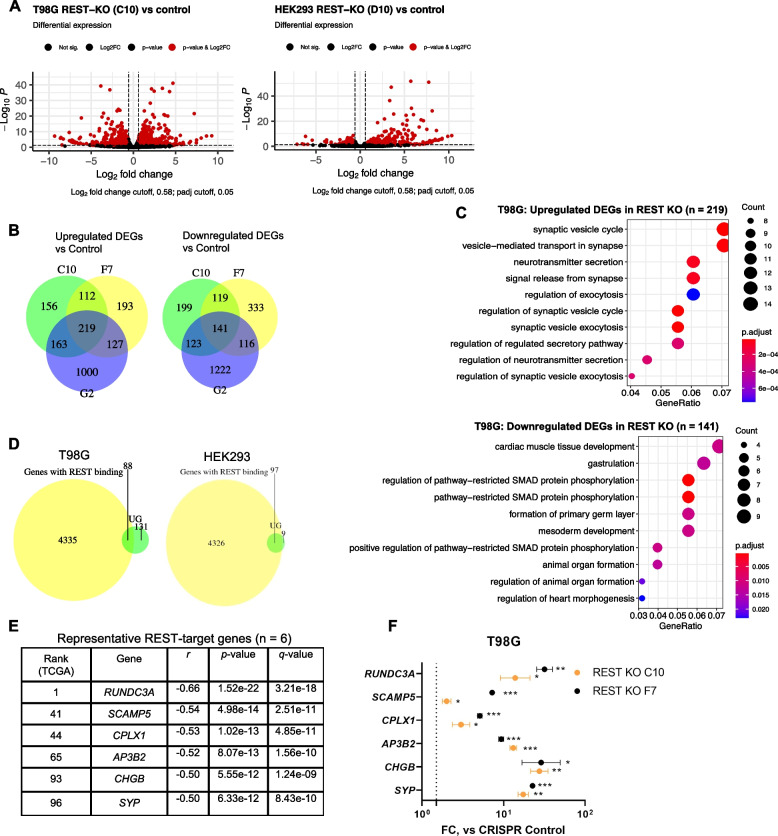
Transcriptome sequencing reveals distinct changes in gene signatures associated with REST.** A** RNA-seq analyses showing gene upregulation and downregulation (highlighted in red, log_2_FC cutoff = 0.58, *p*adj < 0.05) in REST KO cells compared to corresponding control (T98G—*left*, HEK293—*right*). Representative volcano plots were built using “Enhanced Volcano” Bioconductor package. **B** Venn diagrams of deregulated genes shared between three “slow” T98G REST-KO clones (C10, F7, and G2): upregulated genes are shown on the left, downregulated genes are shown on the right. **C** GO (gene ontology) categories enriched among upregulated genes (*top*) and downregulated genes (*bottom*) in T98G REST-KO vs control. **D** Overlap of upregulated genes in glioblastoma (*left*, shared between three “slow” clones, in green) and HEK293 (*right*, shared between two clones, in green) and a published subset of genes with REST binding sites in human embryonic stem cells (in yellow) [29]. **E** A list of representative REST-target genes (*n* = 6) based on Tag-Seq and TCGA-GBM data analysis (shown are correlation coefficients of gene expression with REST mRNA). **F** Validation of REST-target genes by qPCR assay in REST-KO cells. Shown are fold changes (FC) vs T98G control cells derived from three independent biological replicates. Gene expression was measured using ddCt method and normalized by *ACTB* expression. Comparison vs control was performed using one-tailed *t*-tests. Dashed line indicates FC = 1.5. Individual data values are provided in Additional File 10G

To understand if our lead compound could reduce the functional activity of REST in vitro, we exposed human GBM cells to the compound to evaluate its effects on REST-mediated transcriptional silencing. First, to identify REST-controlled genes, we performed Tag-Seq [26] in WT cells and corresponding REST-null clones (Fig. 3A-B, Additional File 1: Fig. S5A-B) in both T98G and non-neural HEK293 cells. Initially, three “slow” REST-KO T98G clones (C10, F7, G2) and two REST-KO HEK293 clones (D10, E6) were sequenced. Sequencing was performed in duplicates (Additional File 1: Fig. S5B), and differentially expressed genes (DEGs) were called for every unique clone versus the corresponding control cell line using DESeq2 (log_2_FC cutoff = 0.58, *p*-adjusted cutoff = 0.05). Absolute numbers and full lists of DEGs can be found in Additional File 3: Table S2 (N1-4, 8–9). Additional File 1: Fig. S6 shows qPCR validation of transcriptomic changes revealed by Tag-Seq. For instance, REST loss in T98G cells led to a reduction in *NEDD9* expression, a marker of glioma invasion potential [27], and an increase in *BEX1* expression, a tumor suppressor gene in malignant glioma [28].

We focused on differentially expressed genes overlapping in several single-cell clones within one cell line (Additional File 3: Table S2, N5,10, Fig. 3B, Additional File 1: Fig. S7A). Unlike in non-neural HEK293 cells, downregulated genes common in T98G REST-KO clones formed several significant gene ontology (GO) categories, highlighting tissue-specific functions of REST in glioblastoma (Fig. 3C**,** *lower*). These data suggest that targeting REST will inhibit several GBM-related pathways [18, 19, 30, 31] in contrast with non-neural cells.

As expected, since REST is a transcriptional silencer, direct REST target genes could be found among upregulated genes that became derepressed after REST perturbation. Consistent with known REST functions [19], GO analysis showed that derepressed genes in both T98G and HEK293 were related to neuron-specific activities such as synaptic vesicle cycle, exocytosis, and neurotransmitter secretion (Fig. 3C, *upper*, Additional File 3: Table S2, N6,11, Additional File 1: Fig. S7B). To identify direct REST targets, we overlapped derepressed genes with a recently published dataset that included genome-wide REST binding sites in human embryonic stem cells [29]. As a result, only 40% of derepressed genes in T98G and the absolute majority of derepressed genes in HEK293 (97/106) were estimated as direct targets of REST (Fig. 3D). REST-target genes common for both cell lines (*n* = 33, Additional File 3: Table S2, N12) were listed as representative REST-controlled genes regardless of cell type.

To identify the genes subject to REST control in GBMs, we prepared a list of representative REST-target genes (*n* = 6) using both the top 100 anti-correlated with REST mRNA genes in TCGA-GBM dataset and our Tag-Seq data overlapping in T98G and HEK293 cells (Fig. 3E). Indeed, all the genes from the list were additionally validated by qPCR assay in two glioblastoma REST-KO clones (Fig. 3F, Additional File 1: Fig. S8B). Interestingly, the majority of included genes were significantly derepressed under REST knockdown in other cancer types, such as endometrial adenocarcinoma, GSE150254 [32, 33], and breast cancer, GSE173857 [34, 35] (Additional File 4: Table S3), suggesting they could be further validated as REST-controlled genes in other cancer types with known REST deregulation.

### Lead compound, GR-28, reduces the transcriptional activity of REST and is cytotoxic against high-REST GBM cells

Since biochemical assays indicated potent in vitro activity of GR-28 against the SCP1 protein, we further sought to assess the lead compound’s effect on the REST protein level and transcriptional activity of REST in the cellular setting.

**Fig. 4:**
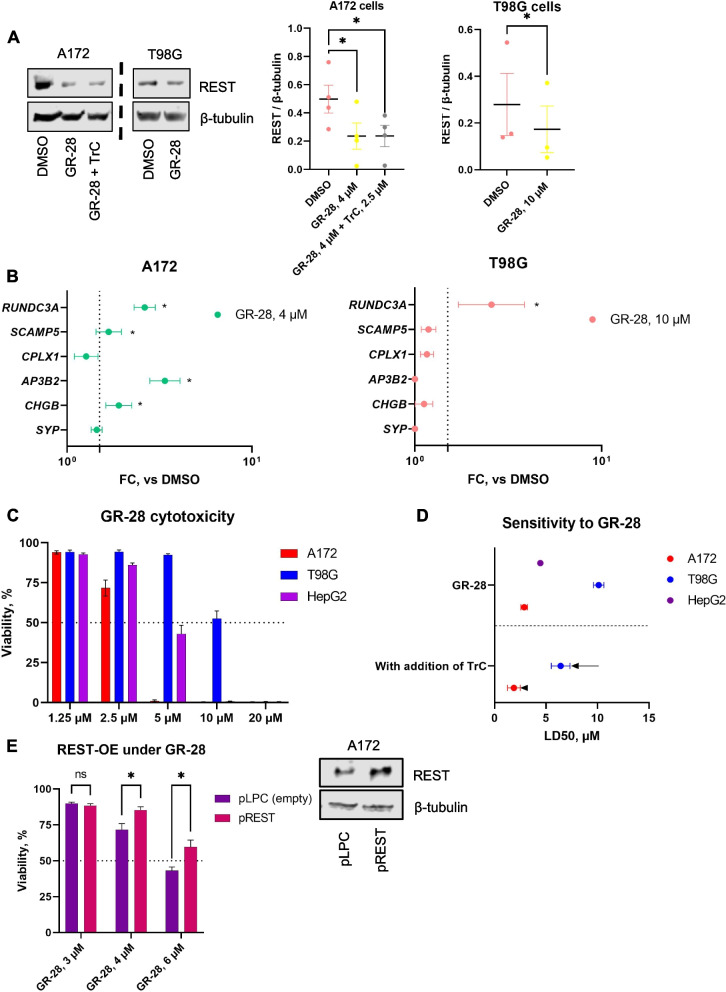
Top lead GR-28 decreases functional activity of REST and is cytotoxic against high-REST GBM cells. **A** Effect of GR-28 on REST protein level in A172 cells (4 μM for 24 h) and T98G cells (10 μM for 48 h). Shown is one representative WB replicate (*left*) and quantification (*right*) from three to four independent cell treatments (mean ± SEM). Comparison vs DMSO was performed using paired one-tailed *t*-tests. Dashed lines indicate that adjacent blots were processed on different days. Individual data values are provided in Additional File 10H-I. **B** Effect of GR-28 on mRNA level of REST-target genes in A172 cells (4 μM for 18 h, *left*) and T98G cells (10 μM for 36 h, *right*). Shown are fold changes (FC) vs DMSO derived from three to four independent biological replicates. Gene expression was measured using ddCt method and normalized by *ACTB* expression. Comparison vs DMSO was performed using paired one-tailed *t*-tests. Dashed line indicates FC = 1.5. Individual data values are provided in Additional File 10J**-**K. **C** Survival rates (72 h) of high-REST GBMs (A172 and T98G) and control cells (HepG2) under single drug treatment with GR-28. Shown are viability rates (mean ± SEM) normalized to that of solvent-control wells derived from three to four independent experiments, *n* = 9–18. **D** Sensitivity (72 h) of high-REST GBMs (A172 and T98G) and control cells (HepG2) to GR-28. Shown are LD50s (lethal doses 50) with 95% confidence intervals calculated from three to four independent biological replicates using “drc” R package. TrC = Triacsin C (sensitization at 0.625 and 2.5 μM in T98G and A172, respectively). **E** Protective effect of REST-OE under GR-28 treatment (24 h) in A172 cells (two independent replicates combined, *n* = 6). Group comparison was performed using multiple *t*-tests. Validation of transient REST overexpression is shown on the right side. *p < 0.05, ns not significant

Western blotting showed that adding 4 µM GR-28 resulted in a 2.1-fold decrease in REST amount in A172 cells after 24 h of incubation (Fig. 4A). This is a big improvement compared to the parental compound, T-65, which could not reduce REST protein levels in GBM cells in the same treatment setting (Additional File 1: Fig. S4D). Furthermore, GR-28 treatment (4 µM, 18 h) of A172 cells significantly increased the transcriptional activity of 4 genes (*RUNDC3A*, *SCAMP5*, *AP3B2*, *CHGB,* FC > 1.5, *p* < 0.05) out of 6 genes that we identified as REST-controlled (Fig. 4B, *left*). As T98G cells were more resistant to GR-28, we applied a longer treatment (48 h) with the compound at a 10 µM dose, which caused a 1.6-fold reduction in REST protein (Fig. 4A). Treatment for 36 h at 10 µM dose markedly increased expression of the *RUNDC3A* gene (FC > 1.5, *p* < 0.05), but expression of other REST-controlled genes did not change significantly (Fig. 4B, *right*).

Next, we analyzed the survival rates of high-REST GBM cells treated with GR-28 at different doses and plotted viability normalized to cells treated with DMSO control (Fig. 4C). We also treated the HepG2 control cell line for a parallel estimation of compound-induced hepatotoxicity [36]. Dose–response fitting showed that the LD_50_ of GR-28 was lower than that of the parental compound T-65: 2.9 µM and 10.1 µM in A172 and T98G cells, respectively (Fig. 4D, *upper,* Additional File 1: Fig. S4C, Additional File 5: Table S4). For comparison, the LD_50_ of T-65 was estimated to be 3.9 µM and 12.5 µM in A172 and T98G cells, respectively. GR-28 affected control cells with an LD_50_ of approximately 5 µM (Fig. 4D, *upper*). Both small molecules, parental T-65 and optimized GR-28, were predicted to be able to cross the blood–brain barrier (BBB) using a recently published deep neural network-based model for the prediction of BBB permeability of novel compounds [37] (Additional File 6: Table S5).

T98G cells were markedly more resistant to SCP1 inhibitors than A172 cells. Notably, protein level of SCP1 was comparable between A172 and T98G cells (Additional File 1: Fig. S4E); hence, it cannot explain higher resistance of T98G cell line. However, T98G cells are generally more resistant to chemotherapeutic drugs than other GBM cell lines [38, 39]. It is likely that resistance of T98G is related to their having specific somatic mutations in tumor suppressor genes (*TP53* M237I, *PTEN* L42R) based on the analysis of COSMIC (Catalogue Of Somatic Mutations In Cancer) database (Additional File 7: Table S6). *TP53* M237I (c.711G > A) point mutation is known to result in decreased DNA binding and apoptotic potential, resistance to cytotoxic agents, and lack of G1 cell cycle arrest [40]. In addition, we and others [41] showed that T98G cells over-express *MGMT* (O^6^-methylguanine methyltransferase) that contributes to their chemoresistance. GR-28 did not change *MGMT* expression (Additional File 1: Fig. S4F).

Finally, to assess the specificity of REST targeting by GR-28 top lead, we transiently transfected more sensitive A172 cells with the plasmid expressing REST (pREST) or empty vector (pLPC) and treated the cells with concentration range of GR-28 (Fig. 4E). Cytotoxicity measurements showed partial rescue effect of REST overexpression, thereby highlighting on-target specificity of GR-28 induced GBM cell death.

These data demonstrate specific and functional REST-inhibitory effects of the top lead, GR-28, on high-REST GBM cells. However, the compound dosage needed to thwart GBM cell growth is still high.

### REST-null GBM cells can rescue their growth through lipid metabolism rewiring

**Fig. 5:**
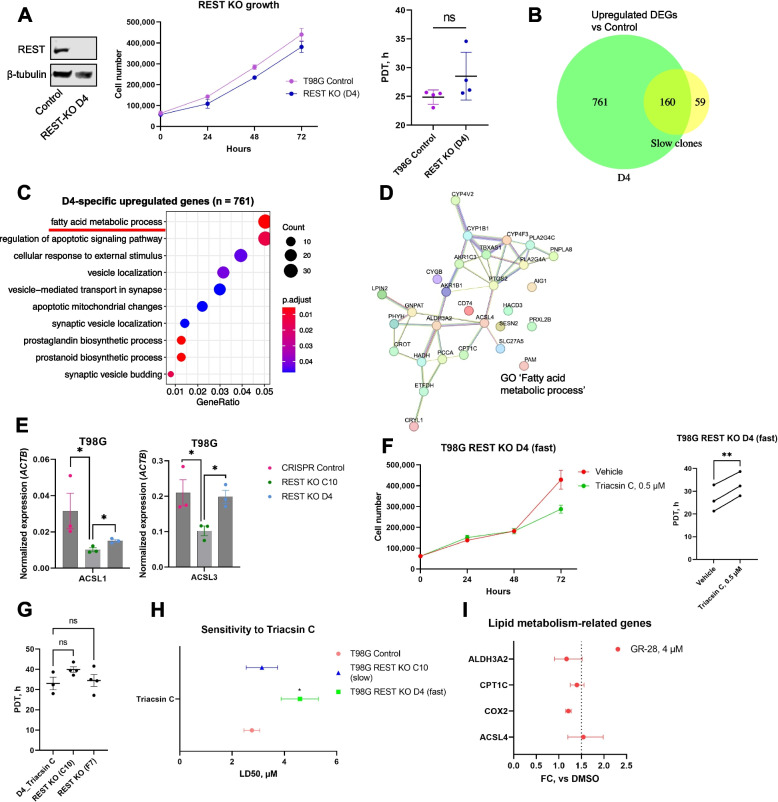
REST-null GBM cells can rescue their growth through lipid metabolism rewiring. **A** *Left*, Western blotting confirms lack of REST protein in D4 cells. Proliferation of WT and D4 cells was examined by counting cells every 24 h. Shown is one representative replicate and quantification of PDT (*right*) based on three to four independent experiments (mean ± SD). Statistical comparison vs T98G control was performed using ANOVA with post hoc tests. Individual data values are provided in Additional File 10C. **B** Venn diagram showing overlap between upregulated DEGs in “slow” clones and “fast” D4 cells. **C** Gene ontology categories significantly enriched among D4-specific upregulated DEGs. **D** Gene network for “Fatty acid metabolic process” (GO:0006631) as a top-significant category from **C**. Network was built using STRING v.12 database. **E** Gene expression of *ACSL1* and *ACSL3* in T98G WT, “slow”, and “fast” REST-KO cells. Statistical difference between groups was tested using *t*-test. Shown are mean ± SEM from three biological replicates. Individual data values are provided in Additional File 10L. **F** Proliferation of D4 cells, in the presence of DMSO or pan-ACSL inhibitor Triacsin C (500 nM) in the media, was examined by counting cells every 24 h. Shown is one representative replicate and quantification of PDT based on three independent experiments. Statistical difference between groups was tested using two-tailed paired *t*-test. Individual data values are provided in Additional File 10 M. **G** Proliferation of D4 cells in the presence of Triacsin C (500 nM) does not differ from that of “slow” REST-KO clones. Comparison was performed using ANOVA with post hoc tests. Individual data values are provided in Additional File 10N. **H** Sensitivity of T98G WT and REST-KO cells to pan-ACSL inhibitor Triacsin C (72 h). Shown are LD50s with 95% confidence intervals calculated from three to four independent biological replicates. LD50s were compared vs T98G control using ratio test from “drc” R package. **I** Effects of GR-28 lead (4 μM for 18 h) on expression of select lipid metabolism genes from the network (**D**). Shown are fold changes (FC) vs DMSO derived from four independent biological replicates. Dashed line indicates FC = 1.5. Individual data values are provided in Additional File 10O. **p < 0.01; *p < 0.05; ns—not significant

Contrary to our observation of REST-null GBM cells having growth arrest, we noticed that some REST-KO GBM clones (e.g., T98G REST-KO D4) still divided at the same rate as control cells even when REST protein was absent (Fig. 5A). Indeed, it has been shown that the integration of mutations into the genome sometimes leads to compensation with little to no effect on cellular phenotype [42]. We speculated that targeting the underlying compensatory pathway in D4 cells might have a synergetic effect with REST inhibition. To identify what kind of adaptation enhanced the growth of these “fast” cells upon REST loss, we compared gene expression in the D4 clone vs three “slow” clones (REST-KO C10, F7, G2) using whole-transcriptome sequencing. First, we overlapped upregulated DEGs shared between “slow” clones and upregulated DEGs identified for the D4 clone (Additional File 3: Table S2, N5,13, Fig. 5B) and found that the majority of shared genes (73%) were also elevated in D4 cells, indicating underlying universal changes induced by REST-KO regardless of cellular phenotype. However, 761 upregulated genes did not overlap with the common “slow” gene set, were specific to the D4 clone, and clustered into several GO categories, the most significant of which was “Fatty acid metabolic process” (*q*-value = 0.005) (Additional File 3: Table S2, N14-15, Fig. 5C). This category is shown as a gene network based on protein–protein interactions in Fig. 5D. As shown in the gene network, the “fast” phenotype was co-occurring with the upregulation of metabolic enzymes such as *CPT1C*, *CROT*, and *PTGS2 (COX-2)*, all of which are oncogenic [43–45].

An important enzyme in the “Fatty acid metabolism” network was *ACSL4*, acyl-CoA synthetase long-chain family member 4 (Fig. 5D). ACSLs (ACSL1, 3, 4, 5, and 6) are a family of CoA synthetases that activate long-chain FAs into acyl-CoA for the synthesis of cellular lipids and are thought to affect cell proliferation, including in nervous system diseases [46]. It is currently known that actively proliferating cancer cells often activate de novo fatty acid synthesis to provide essential structural components, e.g., structural lipids, for their growth [47]. Interestingly, high expression of *ACSL4* positively correlated with cell survival and proliferation after REST perturbation, based on results of pan-cancer RNAi screen by the DepMap project [48] (Additional File 8: Table S7). ACSL1 specifically was reported to be associated with a shorter survival time in GBM patients, and ACSL1 inhibitors could reduce GBM tumor growth both in vivo and in vitro [49]. Based on transcriptome sequencing, expression levels of *ACSL1* were downregulated in all “slow” clones (Additional File 3: Table S2, N5) and significantly correlated with PDT (*r* =  − 0.818, *p* = 0.0038) across all sequenced cells: T98G control, REST-KO C10, F7, G2, D4 (Additional File 3: Table S2, N16, *highlighted*). In addition, a qPCR assay confirmed that *ACSL1* and *ACSL3* mRNA levels were lower in “slow” C10 cells compared to control and were increased in “fast” cells compared to C10 (Fig. 5E).

We thus asked if inhibition of fatty acid metabolic compensation could reverse the “fast” phenotype of T98G D4 cells (Fig. 5F-G). To this end, we turned to the pan ACSL-inhibitor Triacsin C (TrC), a pharmacological intervention that has been shown to prevent lipid accumulation, including in glial cells [50]. Triacsin C directly inhibits ACSL1, ACSL3, and ACSL4 by competing with fatty acids for their catalytic domain [51]. Adding as low as 500 nM of the compound to the cell culture media dramatically increased the proliferation doubling time relative to vehicle-treated D4 cells (Fig. 5F, *right*). However, this dose (500 nM) did not result in a profound decrease in D4 cell viability (> 90%). Triacsin C reduced the proliferation rate of D4 cells to that of “slow” REST-KO clones (Fig. 5G). Viability assays showed that D4 cells were more resistant to Triacsin C-induced toxicity than T98G WT or “slow” REST-KO cells (Fig. 5H). Finally, we selected several important fatty acid metabolism-related genes from the D4 network (Fig. 5D) and measured their activity under treatment with GR-28 (same condition as for REST-target genes expression) (Fig. 5I). qPCR experiments showed that mRNA of all included genes (*ACSL4, ALDH3A2, CPT1C, COX-2*) got somewhat increased after 18 h of treatment (GR-28, 4 µM), but only *ACSL4* gene had reached FC > 1.5 vs DMSO, highlighting the importance of ACSL-enzymes in GBM compensation phenotype and therapy resistance. Overall, these results establish that glioblastoma cells can compensate for the loss of REST required for their growth via upregulation of fatty acid metabolism, specifically, upregulation of ACSL enzymes, which can be blocked with Triacsin C.

### GR-28 lead exhibits profound synergy with ACSL inhibitor in high-REST GBM cells

**Fig. 6:**
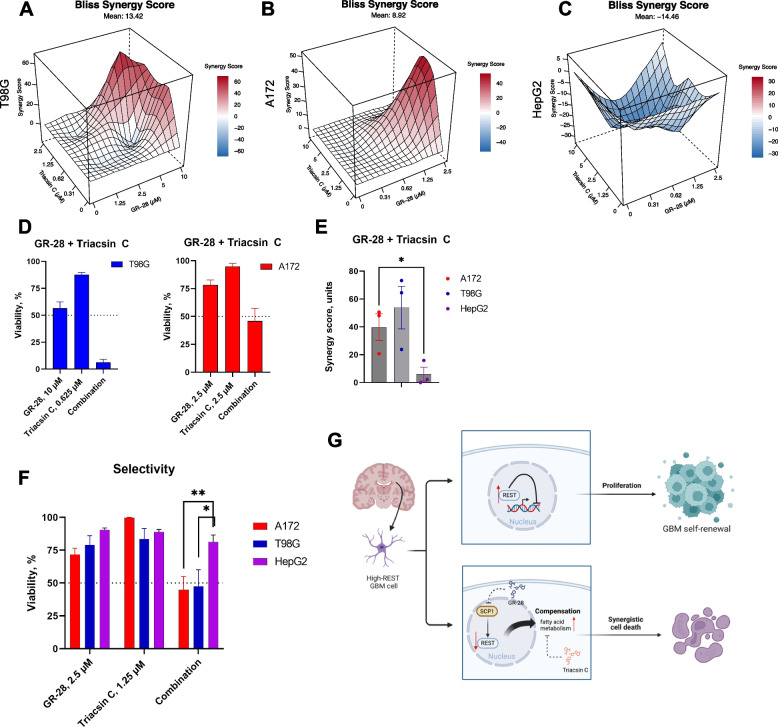
GR-28 exhibits synergy with fatty acid metabolism inhibitor in high-REST GBM cells. **A–C** Drug combination landscapes (72 h) in GBMs (**A–B**) and HepG2 cells (**C**). HepG2 cells were treated under the same doses as A172. Shown is the representative landscape having maximal synergy score closest to its mean value. Landscapes were built using “synergyfinder” R package (Bliss model). **D** Low-toxic dose of Triacsin C sensitizes high-REST GBM cells to GR-28. Shown are viability rates (mean ± SEM) normalized to that of solvent-control wells derived from three independent experiments, *n* = 9. **E** Maximal synergy scores (mean ± SEM) extracted from three independent drug combination landscapes (GR-28/Triacsin C) in A172, T98G, and HepG2 cells. Statistical difference was tested using a *t*-test. Individual data values are provided in Additional File 10P. **F** Selective effect of GR-28/Triacsin C drug combination on GBM cells. Plotted are viabilities (mean ± SEM, 3 independent experiments, *n* = 9–12, 72 h) normalized to that of solvent-control wells under single drug treatments and combination treatments. Statistical difference was tested using multiple *t*-tests. **G** Simultaneous targeting of REST and fatty acid metabolism results in synergistic cell death in GBM cells. Model was created using BioRender software. **p < 0.01; *p < 0.05

We turned to combination anticancer therapy to lower effective doses of GR-28 in GBM cells without severe compound-induced hepatotoxicity. The rationale design of combination drug regimens is a powerful and comprehensive strategy for targeting cancer cells while sparing normal cells [52]. First, we noted that REST knockout led to the activation of genes related to fatty acid metabolism, and then we observed that the addition of pan-ACSL (acyl-CoA synthetase long-chain) inhibitor Triacsin C was able to suppress this compensatory event in REST-KO glioblastoma cells. Therefore, we next tested whether Triacsin C can sensitize wild-type high-REST GBM cells to the top lead, GR-28 (Fig. 6A-B).

For combinatorial treatment, we prepared three serial twofold dilutions from the maximal dose of the single compounds that corresponded to average viability rates > 40–50% in GBM cells based on preliminary single-drug treatments (Fig. 4C, Additional File 1: Fig. S9A*, left*). Since we observed substantial differences in sensitivity to GR-28 and Triacsin C in A172 and T98G cells, individual dose ranges were applied. Drug combinations were used to treat GBM cells in plates for 72 h, along with vehicle-only controls (media with drugs was also renewed daily). The resulting cell viabilities from 5 × 5 matrices based on this multi-ray design [53] were used as input to build a synergy landscape using a Bliss model in the “synergyfinder” R package [54]. Then, maximal synergy scores were determined for each cell line from at least three independent experiments, and the averages were recorded. Hepatocarcinoma cells (HepG2) were treated at the doses corresponding to the A172 glioblastoma cell line, as the one more sensitive to GR-28. We observed that GR-28/Triacsin C combination was highly synergetic against A172 and T98G cells (Fig. 6A-B, Additional File 1: Fig. S9C), but predominantly antagonistic in liver cancer cells (Fig. 6C, Additional File 1: Fig. S9C). Low concentrations of Triacsin C (0.625–2.5 µM) markedly sensitized GBM cells to GR-28, decreasing their LD_50_s 1.5–1.6-fold on average (Fig. 6D, Fig. 4D, *lower*, Additional File 5: Table S4). Despite the differences in T98G and A172 genotypes, the tested drug combination was synergetic against both cell lines. Predictably, adding Triacsin C (2.5 µM) to GR-28 treatment did not further reduce the REST protein amount in A172 cells (Fig. 4A). Importantly, the GR-28/Triacsin C combination had significantly lower synergy scores in liver cancer cells (Fig. 6E), allowing higher selectivity and a significant therapeutic window between GBM cell lines and HepG2 (Fig. 6F). When treated with a single GR-28 compound, T98G cells were 2.3-fold more resistant to GR-28 than HepG2. Still, adding a low dose of Triacsin C could surprisingly reverse this sensitivity pattern (Fig. 6F). As another control, non-cancerous cells of different tissue origin (HEK293) did not exhibit synergy between GR-28 and Triacsin C, mean synergy scores were 5 units or below (Additional File 1: Fig. S10A-C).

As a proof of concept, we also tried a combination of GR-28 with another fatty acid metabolism inhibitor, perhexiline maleate (PER), a potent inhibitor of CPT1/2 [55]. Despite observed synergy in GBM cells (Additional File 1: Fig. S9B), this drug pair is less translationally promising than GR-28 + TrC due to high hepatotoxicity of perhexiline shown in this (Additional File 1: Fig. S9A, *right*) and other [55] studies.

Our results perfectly align with recent suggestions that dysregulated lipid metabolism can contribute to tumor resistance and novel combination therapy strategies can re-sensitize cancer cells to chemotherapy [56]. For instance, paired combinations of Triacsin C with classic chemotherapeutic drugs such as cisplatin, doxorubicin, and paclitaxel resulted in remarkable synergistic anti-tumor effects [57].

## Discussion

In this study, we utilized a combination of genetic and cellular approaches to demonstrate that REST significantly promotes glioblastoma proliferation, as well as plays an important role in GBM cell migration and self-renewal. Our newly designed SCP1 inhibitor GR-28 can reduce REST transcriptional activity by targeting REST for degradation and thus thwart the growth of GBM cells. Intriguingly, we noticed that GBM cells could compensate for the loss of REST via upregulating fatty acid metabolism. Remarkably, our results revealed that combinatorial REST targeting using GR-28 and the fatty acid pathway inhibitor Triacsin C led to synergistic cell death in glioblastoma cells with high basal REST levels (Fig. 6G). This drug combination exhibited limited hepatotoxicity, as it induced little adverse effect on human hepatocarcinoma cells. These findings warrant further investigation of the therapeutic potential for drug combinations targeting REST and lipid metabolism in xenograft GBM models.

The underlying genetic background of the tumor is crucial to the phenotypic heterogeneity and adaptability of glioblastoma [58]. Recent advances in single-cell technologies, particularly single-cell transcriptomics (scRNA-Seq), offer promising avenues for a deeper understanding of intratumor heterogeneity when tailoring personalized cancer therapies [59]. Specifically, glioblastomas with a high-REST tumor subpopulation can be potentially diagnosed and then targeted with selective REST inhibitors alone or combined with other chemotherapeutic agents. Since REST serves as a growth promoter in GBM, targeting REST can multifacetedly impact various signaling pathways for more efficient GBM eradication [3].

Given the significant role of phenotypic plasticity in cancer treatment resistance, especially in GBMs, it became obvious that targeting plasticity and its regulators is crucial to restrict the adaptive capacities of GBM [58]. While some GBM cells may already exist in highly resistant states, “persister” cells can activate various adaptive mechanisms upon treatment, such as entering quiescence or adopting stemness pathways [58]. To address the adaptive capacities of tumors, two major strategies can be used to enhance resensitization to targeted therapy. First, tumor cells are subject to chemotherapy treatment, and then resulting phenotypic changes are recorded with subsequent identification of adaptive tumor response [52, 56]. The second strategy entails using a genetic perturbation, such as knockdown or knockout, as demonstrated in this study. By targeting tumor adaptive responses, combination therapy regimens can augment the treatment efficacy and curb cancer resistance [60, 61].

As we observed in this study, the SCP1 inhibitor GR-28 exhibited limited lethality against GBM cells when used as a single drug, despite its superior specificity. However, this limitation was effectively overcome by combining GR-28 with a pan ACSL inhibitor, which resulted in a significant resensitization to GR-28 while sparing hepatocarcinoma cells. At the same time, specific mechanisms linking REST perturbation and fatty acid metabolism reactivation are yet to be studied. One may hypothesize that specific REST target(s) that become derepressed are actually “gearing up” the GBM cells for fatty acid uptake, storage or use to meet tumor’s increased energy demands. Targeting key lipid metabolism enzymes (SCD, FADS2, ACLY, and ACSL) to re-sensitize cancer cells to chemotherapy has already been demonstrated as a successful approach to combat glioma and glioblastoma [62–64]. Furthermore, in a nude mouse xenograft model, ACSL-inhibitor Triacsin C at a non-toxic dose enhanced the anti-tumor efficacy of low-dose chemotherapy with etoposide, a well-known apoptosis activator [64]. Our results contribute to recent findings regarding the promise of REST inhibition in high REST glioblastoma cells and combination regimens targeting GBM plasticity.

## Conclusions

Reducing REST levels in REST-dependent glioblastoma tumors holds promising therapeutic effects [7]. However, targeting transcription factors with small molecule inhibitors has been challenging [10]. In this study, we hypothesized that the reduction in REST protein levels in high-REST GBM cells could be achieved by regulating its phosphorylation state through the inhibition of C-terminal domain small phosphatase 1 (SCP1). We demonstrated that REST is a driver of glioblastoma proliferation using CRISPR/Cas9 knockout, with genetic analysis also highlighting its critical role in GBM cell migration and stemness maintenance. Having validated oncogenic role of REST in high-REST GBM cells, we rationally designed SCP1 inhibitor GR-28, which reduces REST transcriptional activity, thus thwarting the proliferation of GBM cells. Intriguingly, we observed that REST-null GBM cells could compensate for the loss of REST transcriptional function by upregulating fatty acid metabolism. Exploiting this discovery, we designed a regimen combining GR-28 with an inhibitor of long-chain acyl-CoA synthetases (Triacsin C), resulting in synergistic cell death in high-REST glioblastoma cells. Notably, this drug combination exhibited limited hepatotoxicity and improved selectivity for GBM cells. Future studies will explore the optimal lipid metabolism inhibitors to be coupled with REST inhibitors, including an in vivo setting.

## Methods

### TCGA data mining

TCGA data was analyzed using “TCGAbiolinks” Bioconductor package [65] and GEPIA2 (Gene Expression Profiling Interactive Analysis) web server [66]. REST mRNA expression data (in TPM format, transcripts per million) were compared in TCGA-LGG (low-grade glioma) dataset, TCGA-GBM (glioblastoma) dataset, and matching normal samples from TCGA and GTEx databases via GEPIA2. Survival analysis was performed using the same tool. For prediction of REST-target genes, RNA-Seq data (FPKM-UQ format) was downloaded from the open-access part of the TCGA database for 169 GBM patient samples (Primary Solid Tumor/Recurrent Solid Tumor) on 2/13/2021 using “TCGAbiolinks” R package. After standard pre-processing and filtering of low-signal mRNAs across all samples, pair-wise correlation coefficients between REST expression and expression of every transcript from the remainder (*n* = 42,335) were calculated. Then, the coefficients were ranked in the order of increasing magnitude, and top genes negatively correlated with REST were analyzed in follow-up studies.

### Cell culturing and cell treatments

Human embryonic kidney cells (HEK293/HEK293T), human liver cancer cells (HepG2), human fetal glial cells SVGp12, and glioblastoma cell lines (A172, T98G) were purchased from ATCC (Manassas, VA). U251 glioblastoma cells were purchased from Sigma-Aldrich (St. Louis, MO). HEK293, SVGp12, and GBM cells were routinely cultured in minimal essential media (MEM, Sigma-Aldrich) supplemented with 10% Opti-Gold fetal bovine serum, FBS (GenDEPOT, Katy, TX), 1 mM sodium pyruvate (Sigma-Aldrich), and 1% non-essential amino acids (Sigma-Aldrich) at 37 °C in a humidified 5% CO_2_ atmosphere. HepG2 was cultured in MEM supplemented with 10% FBS. HEK293T cells, CRISPR-edited cell lines, and corresponding CRISPR control cells were cultured in Dulbecco’s modified Eagle’s media (DMEM, Sigma-Aldrich), supplemented with 10% FBS. HyClone penicillin and streptomycin (P/S) mix (Cytiva, Marlborough, MA), at a final concentration of 1%, was added to all media. Prior to the experiments, routinely cultured cell lines were confirmed mycoplasma free by the Mycoplasma qPCR kit (Minerva Biolabs, Skillman, NJ).

Novel small molecule SCP1 inhibitors (T-65, GR-28) were synthesized in Dr. Dionicio Siegel’s lab. Triacsin C/TrC was purchased from Tocris (#2472) or Cayman Chemicals (#10007448). Perhexiline maleate was purchased from MedChemExpress (HY-B1334A). Stock solutions of all the compounds were prepared in DMSO and were stored at − 80°C, avoiding multiple freeze–thaw cycles.

For determination of cytotoxicity, cells at a final density of 8000/well (100 µL) were seeded in black 96-well plates in their corresponding complete media and treated with compound(s) of interest or solvent-control on the next day. For Western blots or RNA extraction, cells were seeded in complete media in flasks and also treated the next day. Wild-type cell treatments were performed for a specified time duration (18, 24, 36, 48, or 72 h) in MEM supplemented with 5% FBS, 1 mM sodium pyruvate, 1% NEAA, and 1% P/S. CRISPR-edited cells and their corresponding controls were treated in DMEM supplemented with 5% FBS and 1% P/S. Drug mixtures with SCP1 inhibitors were replenished daily when treatment duration exceeded 24 h, based on time-course assessment of REST protein recovery under treatment with compounds of this class [14]. DMSO concentrations in the incubation mixtures or solvent-control mixtures never exceeded 0.5% (v/v).

### Design and synthesis of SCP1 inhibitors

The syntheses of a large number of analogues with variable targeted covalent functionality has been possible given the design of our route and generality of the reaction used for the syntheses. Amide coupling reactions with the noncovalent amide region were needed for targeting and provided access to improved compounds. Testing of a large number of reactive warheads with structurally diverse, reactively unique, and positional isomers was used to find the optimal reactive group—benzothiophene-1,1-dioxide [67]. The syntheses followed a similar path as that used for GR-28.

### Synthetic procedures


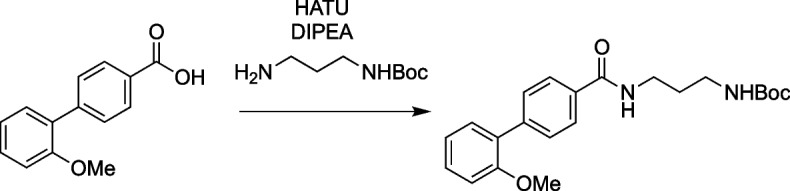


To a stirring solution of 2'-methoxy-[1,1'-biphenyl]-4-carboxylic acid (200 mg, 0.88 µmol, 1.0 equiv) and tert-butyl (3-aminopropyl) carbamate (198 mg, 1.14 mmol, 1.3 equiv) in DMF (5 mL), HATU (666 mg, 1.75 mmol, 2 equiv) and DIPEA (340 mg, 2.63 mmol, 3 equiv) were added, and the reaction was stirred at 23 °C for 14 h. The reaction was then diluted with ethyl acetate (25 mL) and the organic phase was washed with brine (4 × 25 mL). The organics phase was dried over Na_2_SO_4_, concentrated, and purified by column chromatography (1:5 EtOAc:Hexanes) to yield tert-butyl (3-(2'-methoxy-[1,1'-biphenyl]-4-carboxamido)propyl)carbamate (302 mg, 90%).

^1^H NMR (600 MHz, CDCl_3_) δ 7.88 (d, *J* = 7.9 Hz, 2H), 7.60 (d, *J* = 8.2 Hz, 2H), 7.34 (dd, *J* = 17.3, 8.4 Hz, 2H), 7.22 (s, 1H), 7.04 (t, *J* = 7.4 Hz, 1H), 7.00 (d, *J* = 8.2 Hz, 1H), 4.98 (s, 1H), 3.81 (s, 3H), 3.53 (dd, *J* = 12.2, 6.1 Hz, 2H), 3.36–3.18 (m, 2H), 1.72 (s, 2H), 1.46 (s, 9H).

HRMS: m/z: calcd for C_22_H_28_N_2_O_4_: 385.2122; found 385.2120.
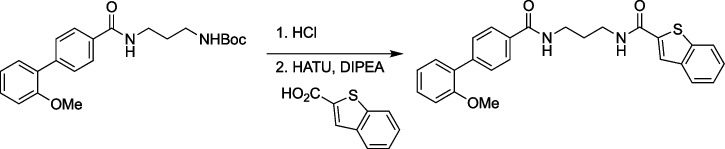


To a stirring solution of tert-butyl (3-(2'-methoxy-[1,1'-biphenyl]-4-carboxamido)propyl) carbamate (270 mg, 702 µmol, 1 equiv) in MeOH (3 mL), concentrated aqueous HCl (15 mL) was added. The solution was stirred at 23 °C for 15 min. Completion of reaction was monitored by TLC and which was subsequently concentrated in vaccuo to afford 3-(2'-methoxy-[1,1'-biphenyl]-4-carboxamido)propan-1-aminium chloride which was used directly for the next reaction. The solid was dissolved in DMF (2 mL) and benzo[b]thiophene-2-carboxylic acid (162 mg, 912 µmol, 1.3 equiv) was added followed by HATU (533 mg, 1.4 mmol, 2 equiv) and DIPEA (272 mg, 2.1 mmol, 3 equiv). The reaction was stirred at 23°C for 14 h and diluted with ethyl acetate (15 mL). The mixture was washed with brine (4 × 15 mL). The organic extract was dried over Na_2_SO_4_, concentrated under vacuum, and purified by column chromatography (1:1 EtOAc: Hexanes) to yield N-(3-(2'-methoxy-[1,1'-biphenyl]-4-carboxamido)propyl) benzo[b]thiophene-2-carboxamide (260 mg, 82%). Rf = 0.5 (silica gel, 0:1 hexanes: EtOAc).

^1^H NMR (600 MHz, CDCl_3_) δ 7.93–7.89 (m, 3H), 7.85 (t, *J* = 8.3 Hz, 2H), 7.62 (d, *J* = 8.1 Hz, 2H), 7.59 (t, *J* = 6.0 Hz, 1H), 7.44–7.33 (m, 3H), 7.31 (d, *J* = 7.4 Hz, 1H), 7.09 (t, *J* = 6.1 Hz, 1H), 7.05 (t, *J* = 7.4 Hz, 1H), 7.00 (d, *J* = 8.2 Hz, 1H), 3.81 (s, 3H), 3.62 (dd, *J* = 11.8, 6.2 Hz, 2H), 3.57 (dd, *J* = 11.7, 6.1 Hz, 2H), 1.88–1.82 (m, 2H).

HRMS: m/z: calcd for C_26_H_24_N_2_O_3_S: 445.1580; found 445.1578.
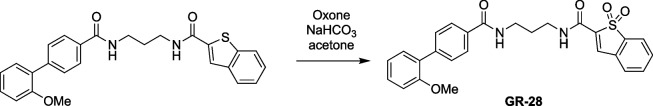


To a vigorously stirred solution of N-(3-(2'-methoxy-[1,1'-biphenyl]-4-carboxamido)propyl) benzo[b]thiophene-2-carboxamide (30 mg, 67 µmol, 1 equiv) in dichloromethane (15 mL), acetone (5 mL) and aqueous, saturated sodium bicarbonate (100 mL) solution were added. Eight portions of oxone (total amount 8.00 g, 13.0 mmol, 190 equiv) were added to this in 5-min intervals. Upon completion, by TLC, the reaction is diluted with water (50 mL) and dichloromethane (20 mL). The layers were separated and the aqueous layer was extracted with dichloromethane (20 mL). The organics were combined, washed with brine (40 mL), dried with MgSO4, filtered, and concentrated in vaccuo. Crude reaction mixture was purified by column chromatography (1:99 MeOH:DCM) to afford N-(3-(2'-methoxy-[1,1'-biphenyl]-4-carboxamido)propyl) cinnamamide 1,1-dioxide (23 mg, 72%). Rf = 0.3 (silica gel, 9.5:0.5 DCM:MeOH).

^1^H NMR (600 MHz, MeOD) δ 7.93 (s, 1H), 7.87 (d, *J* = 8.3 Hz, 2H), 7.80–7.77 (m, 1H), 7.73–7.69 (m, 2H), 7.66 (m, 1H), 7.58 (d, *J* = 8.2 Hz, 2H), 7.35 (t, *J* = 7.8 Hz, 1H), 7.30 (d, *J* = 7.5 Hz, 1H), 7.09 (d, *J* = 8.3 Hz, 1H), 7.03 (t, *J* = 7.5 Hz, 1H), 3.81 (s, 3H), 3.50 (m, 4H), 1.93 (m, 2H).

HRMS: m/z: calcd for C_26_H_24_N_2_O_5_S: 477.1479; found 477.1483.

### Full synthetic scheme for the synthesis of GR-28


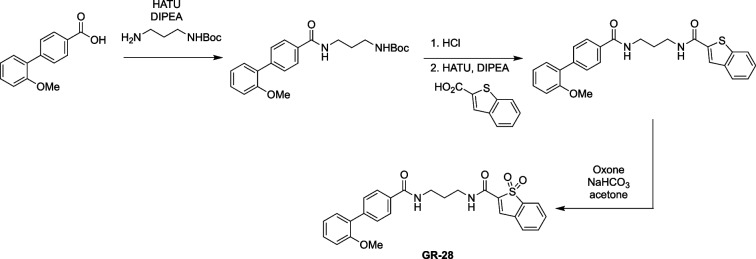


### Kinetic characterization of GR-series compounds

To determine the potency of GR compounds in inhibiting SCP1 phosphatase activity towards the analog substrate *pNPP*, we followed the protocols and methods described in [68–71]. The enzyme concentration was set at 150 nM for SCP1. To study the inhibitory effect, inhibitors were preincubated for varying time durations ranging from 30 min to 23 h at room temperature (RT), and the concentration of DMSO was normalized to 1% in the final reaction volume. In the control group, which did not contain the compound, DMSO was added at a concentration of 1%. The reaction time was set to 3 min at 37 °C. The activity of each phosphatase towards *pNPP* in the presence or absence of the inhibitor was measured in an assay buffer (50 mM Tris–acetate pH 7.6, 10 mM MgCl_2_, 0.02% Triton X-100, and 1% DMSO). The amount of released pNP was quantified by measuring the absorbance at 410 nm. The *k*_*inact*_ and* K*_*I*_ values were calculated using KaleidaGraph software.

### Malachite green assay

Initially, SCP1 was preincubated with either DMSO (as a control) or GR28 (20 µM) for 6/18 h. Subsequently, these samples underwent a 20-min incubation at 37 °C with the different concentrations of physiological substrate, REST phospho-peptide containing phosphorylated serine at position 861. Finally, the reaction was terminated by adding 40 μl of malachite green reagent (Biomol, Hamburg, Germany) to each 20 μl sample. This addition resulted in a green color, the intensity of which is directly proportional to the amount of inorganic phosphate released from pREST during the assay.

### MALDI-TOF analysis of covalent adducts

SCP1 WT (50 μM) and SCP1 C181A mutant in activity buffer at pH 7.6 (with 5 μM or 500 μM BME) were treated with 500 μM of GR-28 (final 1% DMSO), and control samples were treated with 1% DMSO. The samples were incubated overnight at RT. The *pNPP* activity was tested the next day by taking a sample from each tube. The samples were then desalted using Ziptip C18 resins (Sigma-Aldrich) following standard protocols. Mass spectrometric analysis of SCP1 treated with GR-28 or DMSO was performed using an Auto-flex Max MALDI-TOF (Bruker Corporation, Billerica, MA) with a 1:1 DHB matrix (ThermoFisher, Waltham, MA).

### Molecular docking

The model of GR28 compound was built using MAESTRO v. 13.5.128 from the Schrödinger suite. The compound was positioned manually into the active site of SCP1 in PyMOL v. 2.4.1 (PDB Code: 3PGL). Energy minimization of the protein and the manually positioned GR28 compound was performed in MAESTRO by applying the OPLS_2005 force field. The final model was visualized in PyMOL.

### Establishment of CRISPR/Cas9-REST-KO cell lines

To express REST-KO sgRNAs, two pairs of DNA oligos were synthesized: REST-RY1F (caccgGTTATGGCCACCCAGGTAAT) and REST-RY1R (aaacATTACCTGGGTGGCCATAACC); REST-RG6F (caccgGTCTTCTGAGAACTTGAGTA) and REST-RG6R (aaacTACTCAAGTTCTCAGAAGACC) [17]. Annealed double-stranded sgRNAs were cloned into pX330 plasmid [72], and the correct clones were verified by sequencing using U6 promoter primer. Non-neural HEK293 cells and glioblastoma cells T98G were co-transfected with two sgRNA expression vectors (RY1, RG6) and Cas9-2A-GFP plasmid [72] using Fugene^R^ HD (Promega, Madison, WI) transfection reagent according to manufacturer’s guidelines. As a CRISPR-recombination control, we used cells co-transfected with empty pX330 vector and Cas9-2A-GFP plasmid. At 48 h post-transfection, single GFP-expressing cells were sorted using MA900 cell sorter (Sony Biotechnology, San Jose, CA) into 96-well plates (one cell per well) with complete DMEM media for clone expansion. Western blotting was used for screening REST-KO single-cell clones.

### Genotyping of CRISPR/Cas9 repair outcomes

We used Sanger sequencing to identify REST protein sequence in REST-KO clones after double-nicking CRISPR/Cas9 recombination. Briefly, we extracted genomic DNA from target cells using Monarch kit (NEB, Ipswich, MA). Next, we designed primers flanking the regions of sgRNA-guided double-stranded breaks (RY1, RG6) and performed PCR reactions with subsequent Sanger sequencing of PCR products where applicable (Additional File 9: Table S8). For PCR, we used GC buffer and Phusion DNA-polymerase (ThermoFisher) and dNTP mixture from NEB. PCR reactions were run on MasterCycler nexus (Eppendorf, Enfield, CT), and resulting agarose gels were visualized using GelDoc XR + imager (Bio-Rad, Hercules, CA). PCR products were purified using PCR/gel purification kits following manufacturer’s instructions (Qiagen, Germany) and submitted for sequencing with original primer sequences (RY1-F, RY1-R, RG6-F, RG6-R). Sequences were translated into protein using Expasy online tool (www.expasy.org).

### REST transient overexpression

For the rescue experiments, REST-null glioblastoma cells were seeded at 800,000 cells per t-25 flask. On the next day, cells were transiently transfected with 500 ng either REST-WT-expressing pLPC-vector (Addgene, #41903) or empty pLPC vector (Addgene, #12521) with Fugene HD transfection reagent (Promega) following manufacturer’s instructions. At 24 h post-transfection, cells were collected and used for proliferation assay and Western blotting. For the rescue experiments on wild-type glioblastoma cells, transient transfection was performed in 6-well plates with 50 ng of REST-pLPC-vector or empty pLPC-vector per well.

### Western blots

Briefly, cells were lysed in RIPA buffer (10 mM Tris–HCl, pH 7.5, 150 mM NaCl, 1% sodium deoxycholate, 0.1% SDS, 1% Triton x-100, 5 mM EDTA) supplemented with 1 × Halt protease and phosphatase inhibitor cocktail (ThermoFisher) followed by centrifugation for 15 min at 12,000 rpm. Protein concentration in supernatants was measured with BCA assay. Typically, proteins (50 µg) were separated by Novex 4–12% Tris–Glycine gels (Invitrogen, Waltham, MA), transferred to PVDF membranes (Bio-Rad), followed by membrane blocking at room temperature for 1 h in 1% Tween-20-TBS buffer containing 5% BSA (bovine serum albumin, ThermoFisher) or non-fat milk. Membranes were incubated at 4 °C overnight with primary rabbit antibodies against REST (#22242–1-AP, Proteintech, Rosemont, IL) at 1:500 dilution, SCP1 (#ab136038, Abcam, Cambridge, MA) at 1:500 dilution, or *β*-tubulin (#ab6046, Abcam) at 1:4000 dilution. On the next day, membranes were washed and incubated with 1:15,000 diluted goat anti-rabbit secondary IRDye 680RD antibody (LI-COR, Lincoln, NE) for 1 h at room temperature. After washing, membranes were visualized on LI-COR Odyssey CLx image reader.

### RNA isolation, library preparation, and Taq-Sequencing

Total RNA was isolated from cells using DirectZol RNA Miniprep kit (Zymo Research, Irvine, CA, product number #R2050). 3’ Tag-Seq was performed by the University of Texas Genomic Sequencing and Analysis Facility, based on the protocols from Lohman et al. [26] and Meyer et al. [73]. Libraries were quantified using the Quant-it PicoGreen dsDNA assay (ThermoFisher) and pooled equally for subsequent size selection at 350–550 bp on a 2% gel using the Blue Pippin (Sage Science, Beverly, MA). The final pools were checked for size and quality with the Bioanalyzer High Sensitivity DNA Kit (Agilent, Santa Clara, CA) and their concentrations were measured using the KAPA SYBR Fast qPCR kit (Roche, Basel, Switzerland). Samples were then sequenced on the NovaSeq 6000 (Illumina, San Diego, CA) instrument with single-end, 100-bp reads.

### Tag-Seq data analysis

Quality of raw reads was assessed using FastQC read quality reports (https://usegalaxy.org [74]). Adaptor trimming, deduplicating, and quality filtering were performed using a published pipeline (https://github.com/z0on/tag-based_RNAseq). Next, trimmed reads were aligned to human reference genome, GRCh38 version, using HISAT2 fast aligner v.2.2.1 [75] with default parameters, except Forward (F) –rna-strandedness. Gencode v38 gtf file was used as annotation gtf. Lastly, mapped fragments were quantified by featureCounts v.2.0.1 [76] in Galaxy. Differential expression was analyzed using DESeq2 v.1.30.1 [77] in R; genes with adjusted *p*-value < 0.05 and FC cutoff of 1.5 were considered as differentially expressed. Tag-Seq data was deposited in Gene Expression Omnibus/GEO under the accession number GSE234912. GO-enrichment analysis of gene clusters was performed using Bioconductor R package “clusterProfiler” v.3.18.1 [78] and STRING v.12 [79]. For every gene network based on protein–protein interactions (PPI), PPI enrichment *p*-value or FDR was recorded. For correlation with PDT (proliferation doubling time), raw counts were converted to FPKM in R.

### qPCR

Total RNA was isolated from cells using either DirectZol RNA Miniprep kit (Zymo Research) or Trizol reagent with subsequent isopropanol precipitation; 0.5 µg of total RNA was used for reverse transcription using the AzuraQuant™ cDNA Synthesis Kit, #AZ-1995 (Azura, Raynham, MA, USA) using manufacturer’s guidelines. Relative gene expression was measured using AzuraQuant™ Green Fast qPCR Mix, Lo-Rox (Azura) and normalized to *ACTB* gene expression. Amplification was performed using the ViiA 7 Real-Time PCR System (Applied Biosystems, Waltham, MA). Specificity of amplification was controlled with melting curves/primer efficiency calculation. Analysis of qPCR data was performed using the ∆∆Ct method. Primer sequences (designed to span exon-exon junctions or to be separated by a relatively large intron) and qPCR conditions are shown in Additional File 9: Table S8.

### Cytotoxicity assays (resazurin reduction) and drug combination landscapes

After treatment (72 h), 20 µl 0.15 mg/ml resazurin solution (ThermoFisher) was added to each well of a 96-well plate. After 3 h incubation, fluorescence was recorded using a 560-nm excitation/590-nm emission filter set on an Infinite F200 microplate reader (Tecan, Switzerland) [80]. Cell viability was normalized to that of control wells after background subtraction. LD50s of selected compounds were fitted based on cell survival data using the “drc” (dose–response curves) R package [81]. To assess synergetic effects of GR-28 with Triacsin C, we built 5 × 5 drug combination landscapes using the Bioconductor package “synergyfinder” and its Bliss model [54]. For every landscape, cells were seeded in plates and treated the next day with serial twofold dilutions of drug(s), and the maximal dose of single drugs corresponded to 40–50% or higher mean viability. HepG2 cells were treated at the doses corresponding to the A172 cell line which was more sensitive to SCP1 inhibitors, HEK293 were treated at T98G doses. Maximal synergy coefficients were extracted from each landscape and recorded.

### Migration (wound scratch) assay

On day 0, glioblastoma cells were seeded at a density of 400,000 per well in complete media in 6-well plates. On the next day, after reaching 70–80% confluency, cell monolayer was scraped in a straight line with a pipette tip. After scratch, monolayer was gently washed with 1 × PBS to remove detached cells, and media was replenished. Images were taken using EVOS FL fluorescence microscope (Invitrogen) at 4 × magnification 24 h and 48 h post-wound. Images were analyzed using ImageJ software and the Wound_healing_size_tool plugin [82].

### Cell proliferation assay

For the proliferation assay, cells were seeded at a density of 50,000 cells per well in complete media in 24-well plates. Then, cells were counted every 24 h for four subsequent days using Trypan Blue exclusion assay (0.4%) on automated Luna-II automated cell counter (Logos Biosystems, Annandale, VA). Population doubling time (PDT) was estimated with the following formula, PDT = (72 h × ln2) / ln(N_4_/N_1_), where N_1_ and N_4_ are cell counts in every well on first and fourth days, respectively.

### Statistical analyses

Statistical analyses were performed using RStudio v.4.0.5 and GraphPad Prism v.9.5. One-tailed or two-tailed, unpaired or paired (where applicable) *t*-test was used for comparing two groups. ANOVA was used when comparing several groups vs control. *p* < 0.05 values were considered as significant. Correlations were assessed using two-tailed Pearson *r* coefficients. Protein bands were quantified and compared using ImageJ software. Illustrations were created using BioRender software. The statistical details of experiments can be found in the figure legends.

### Supplementary Information

**Additional file 1:** **Fig. S1.** Survival analysis using data from TCGA database. **Fig. S2.** REST-KO using CRISPR/Cas9 gene editing. **Fig. S3.** Tag-Seq analysis of REST-KO cells highlights genes related to regulation of cell migration. **Fig. S4.** T-65 as a top hit from first-generation focused library of SCP1 inhibitors. **Fig. S5.** Tag-Seq analysis of REST-KO cells. **Fig. S6.** Validation of Tag-Seq data using qPCR assay for selected deregulated genes. **Fig. S7.** Transcriptome sequencing in REST-KO HEK293 cells. **Fig. S8.** Assessment of functional level of REST in glioblastoma cells. **Fig. S9.** Sensitization of GBM cells to GR-28 compound by addition of lipid metabolism inhibitors. **Fig. S10.** Sensitivity of non-cancerous HEK293 cells to a combination of GR-28+TrC.

**Additional file 2.** Genotyping of CRISPR/Cas9 repair outcomes.

**Additional file 3.** Tag-Seq analysis in T98G and HEK293 cells: differential expression, gene ontology, overlap analysis.

**Additional file 4.** Analysis of published datasets on REST knockdown in other cell types.

**Additional file 5.** Sensitivity of tested cell lines to SCP1 inhibitors: LD50 values.

**Additional file 6.** Prediction of BBB permeability of novel compounds.

**Additional file 7.** COSMIC database analysis of somatic mutations in A172 and T98G cells.

**Additional file 8.** Depmap data on REST / predictability tab.

**Additional file 9.** Primer sequences used in the study.

**Additional file 10.** Individual data values for plots where *n* < 6.

**Additional file 11.** Original blots.

**Additional file 12.** TCGA database analysis of REST correlations.

## Data Availability

All data generated or analyzed in this study, as well as the code, are included in this published article, its supplementary information files, or publicly available repositories. Any additional information required to reanalyze the data reported in this work is available from the lead contact upon request. Tag-Seq data were deposited in Gene Expression Omnibus/GEO under the accession number GSE234912.

## Abbreviations

*ACSL*: Acyl-CoA synthetase long-chain
*DEG*: Differentially expressed genes
GBM: Glioblastoma
GSC: Glioblastoma stem cells
KO: Knockout
LGG: Low-grade glioma
OE: Overexpression
PDT: Proliferation doubling time
REST: RE-1 silencing transcription factor
SCP1: C-terminal domain small phosphatase 1
TCGA: The Cancer Genome Atlas
TrC: Triacsin C
