# Supplementary material for: Targeting of REST with rationally-designed small molecule compounds exhibits synergetic therapeutic potential in human glioblastoma cells
Source: BMC Biol. 2024 Apr 12;22:83. doi: 10.1186/s12915-024-01879-0 (PMC11015551; doi:10.1186/s12915-024-01879-0)
Supplement: Supplementary file 1 — Additional file 1: Fig. S1. Survival analysis using data from TCGA database. Fig. S2. REST-KO using CRISPR/Cas9 gene editing. Fig. S3. Tag-Seq analysis of REST-KO cells highlights genes related to regulation of cell migration. Fig. S4. T-65 as a top hit from first-generation focused library of SCP1 inhibitors. Fig. S5. Tag-Seq analysis of REST-KO cells. Fig. S6. Validation of Tag-Seq data using qPCR assay for selected deregulated genes. Fig. S7. Transcriptome sequencing in REST-KO HEK293 cells. Fig. S8. Assessment of functional level of REST in glioblastoma cells. Fig. S9. Sensitization of GBM cells to GR-28 compound by addition of lipid metabolism inhibitors. Fig. S10. Sensitivity of non-cancerous HEK293 cells to a combination of GR-28+TrC. [file 12915_2024_1879_MOESM1_ESM.pdf]

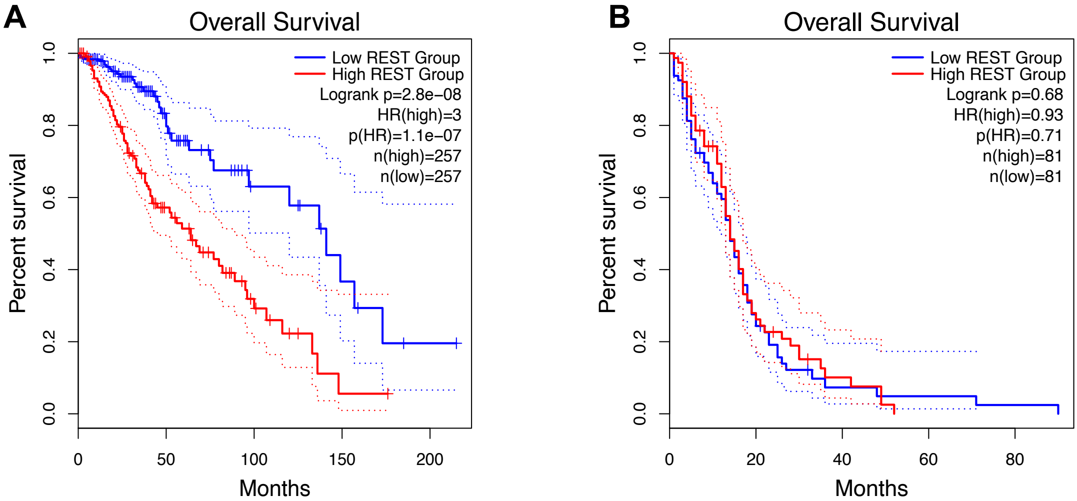

Figure S1. Survival analysis using data from TCGA database: TCGA-LGG (**A**) and TCGA-GBM (**B**) projects. Patient samples were divided into two cohorts: low-REST group (in blue) with REST mRNA level < median; high-REST group (in red) with REST mRNA level > median. Analysis was done using GEPIA2 web server.

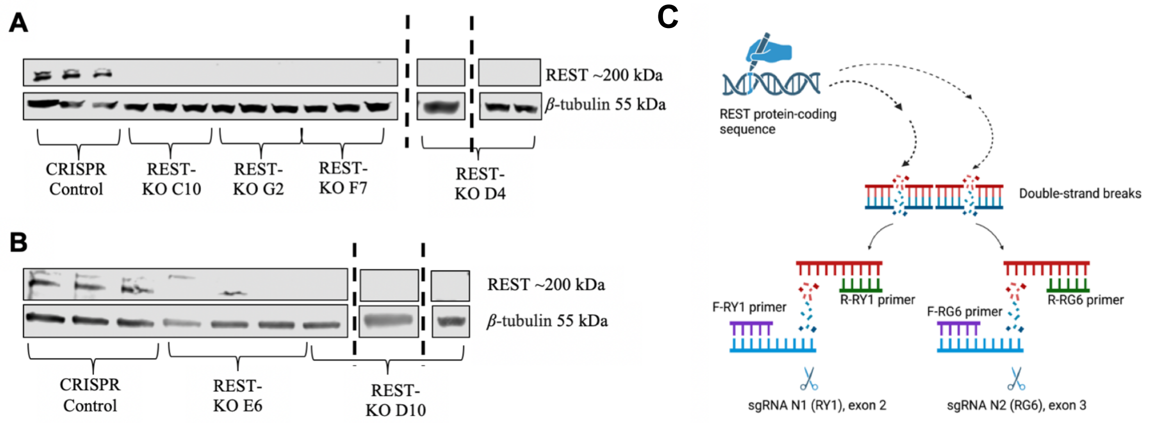

Figure S2. REST-KO using CRISPR/Cas9 gene editing. **A-B,** Western blots confirming absence of REST protein band in REST-KO clones (three independent biological replicates per cell line): **A**, T98G cells. **B**, HEK293 cells. Dashed lines indicate that adjacent blots were processed on different days. **C**, Design of PCR reactions to identify the CRISPR/Cas9-repair outcomes in target cells. Yielded PCR products (where the bands were detected) were purified and analyzed with Sanger sequencing.

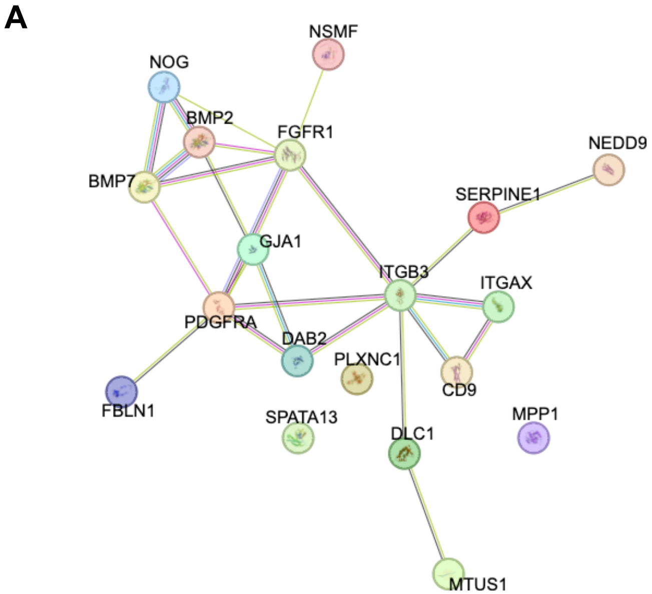

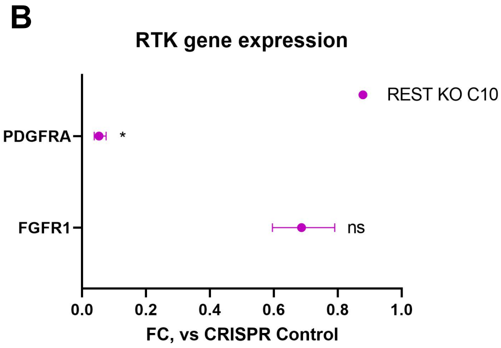

Figure S3. Tag-Seq analysis of REST-KO cells highlights genes related to regulation of cell migration **A,** Gene network (GO:00303334 'Regulation of cell migration') among shared downregulated genes in slow REST-KO clones (C10, F7, G2) vs T98G control based on Tag-Seq results. Gene network based on protein-protein interactions was built using STRING v.12.0 software. **B**, qPCR validation for RTK (receptor tyrosine kinase) expression showed that *PDGFRA* gene expression is strongly inhibited in REST-KO cells. Shown are Fold Changes (FC) vs CRISPR Control derived from 3 independent biological replicates. Gene expression was measured using ddCt method and normalized by *ACTB* expression. Comparison vs Control was performed using unpaired one-tailed t-tests. Individual data values are provided in Additional File 10Q. *$p < 0.05$; ns – not significant.

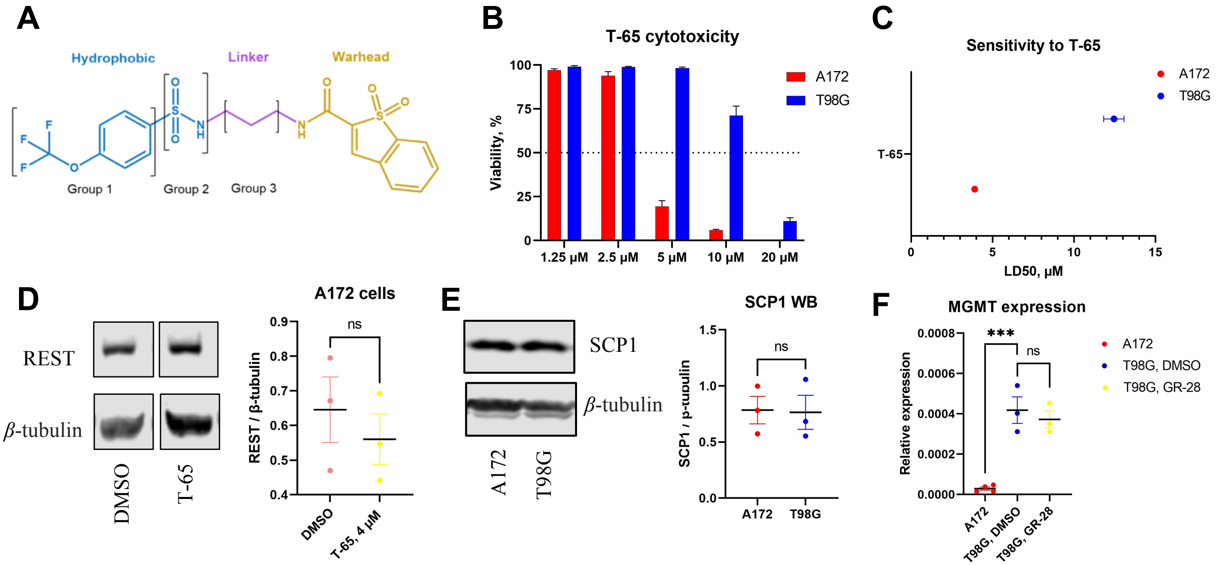

Figure S4. T-65 as a top hit from first-generation focused library of SCP1 inhibitors. **A**, The scaffold of T-65 top lead in T-series sulfone compounds. **B**, Survival rates (72 h) of high-REST GBMs (A172 and T98G) under single drug treatment with T-65 compound. Shown are viability rates (mean±SEM) normalized to that of solvent-control wells derived from 3-4 independent experiments. **C**, Sensitivity (72 h) of high-REST GBMs (A172 and T98G) to T-65. Shown are LD50s (lethal doses 50) with 95%-confidence intervals calculated from 3-4 independent biological replicates using 'drc' R package. **D**, T-65 treatment (4 µM for 24 h) did not change REST protein amount in high-REST glioblastoma cells (A172). Shown are representative blot and mean ± SEM from 3 independent biological replicates. Statistical comparison vs DMSO was performed using one-tailed t-test. Individual data values are provided in Additional File 10R. **E**, A172 and T98G do not differ in SCP1 protein amount on the steady-state level. Shown are representative blot and mean ± SEM from 3 independent biological replicates. Statistical comparison was performed using two-tailed t-test. Individual data values are provided in Additional File 10S. **F**, MGMT gene expression in A172 and T98G cells. Shown are relative expression values normalized by *ACTB* gene expression. Statistical comparisons were performed using t-test. Individual data values are provided in Additional File 10T. ***$p < 0.001$, ns - not significant.

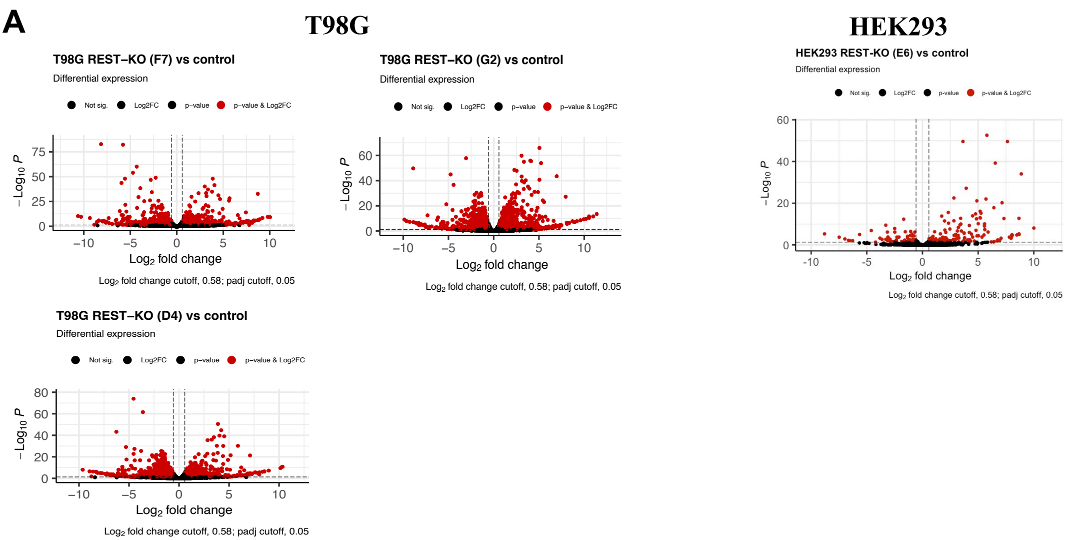

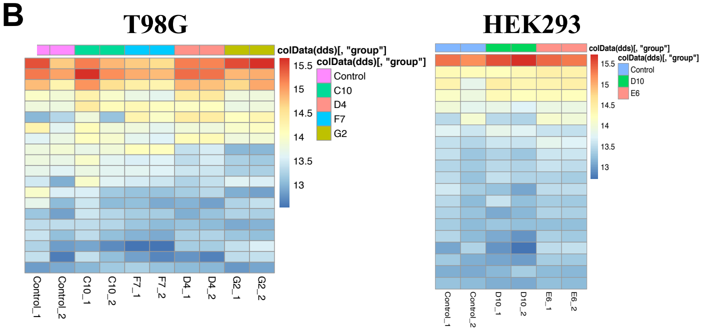

Figure S5. Tag-Seq analysis of REST-KO cells. **A**, RNA-seq shows gene upregulation and downregulation (highlighted in red, log_2FC cutoff = 0.58, padj < 0.05) in REST-KO cells compared to corresponding control. Volcano plots were built using 'Enhanced Volcano' Bioconductor package. **B**, Heatmaps of rlog-normalized RNA-Seq counts across sequenced cell lines (T98G, *left*; HEK293, *right*). Duplicate replicates of every cell line cluster together. Heatmap was built using 'DESeq2' R package.

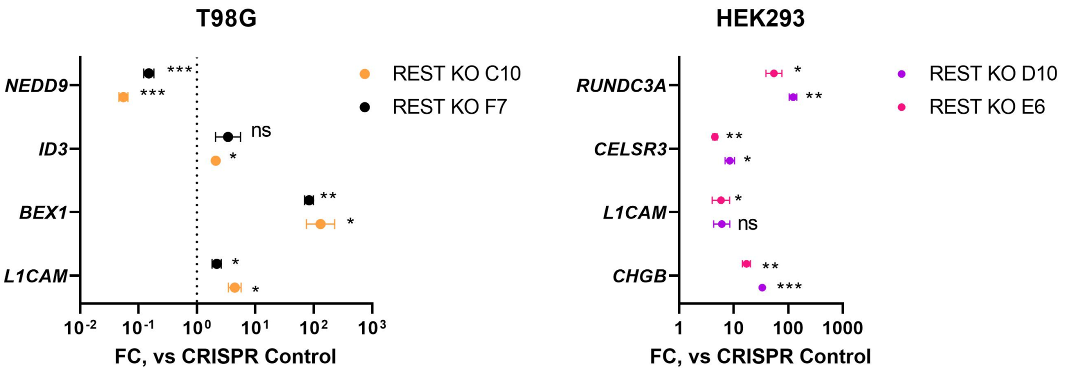

Figure S6. Validation of Tag-Seq data using qPCR assay for selected deregulated genes (T98G, *left*; HEK293, *right*). Shown are Fold Changes (FC) vs respective CRISPR control cells derived from 3 independent biological replicates. Gene expression was measured using ddCt method and normalized by *ACTB* expression. Comparison vs control was performed using unpaired one-tailed t-tests. Individual data values are provided in Additional File 10U-V. ***$p < 0.001$; **$p < 0.01$; *$p < 0.05$; ns – not significant.

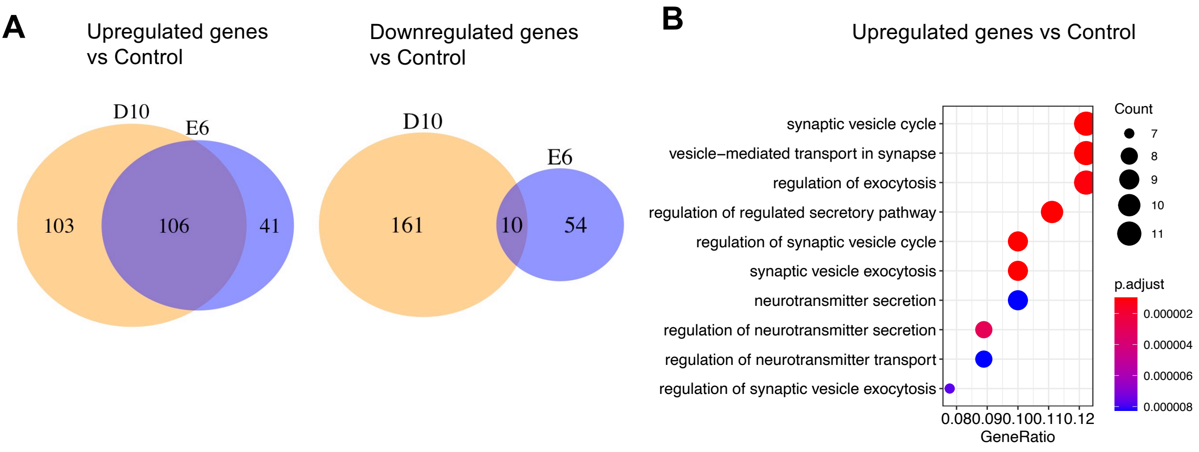

Figure S7. Transcriptome sequencing in REST-KO HEK293 cells. **A**, Venn diagrams of deregulated genes shared between two HEK293 REST-KO clones (D10&E6): upregulated genes are shown on the left, downregulated genes are shown on the right. **B**, GO categories enriched among upregulated genes (n = 106) in REST-KO cells. GO analysis was performed using ‘clusterProfiler’ Bioconductor package.

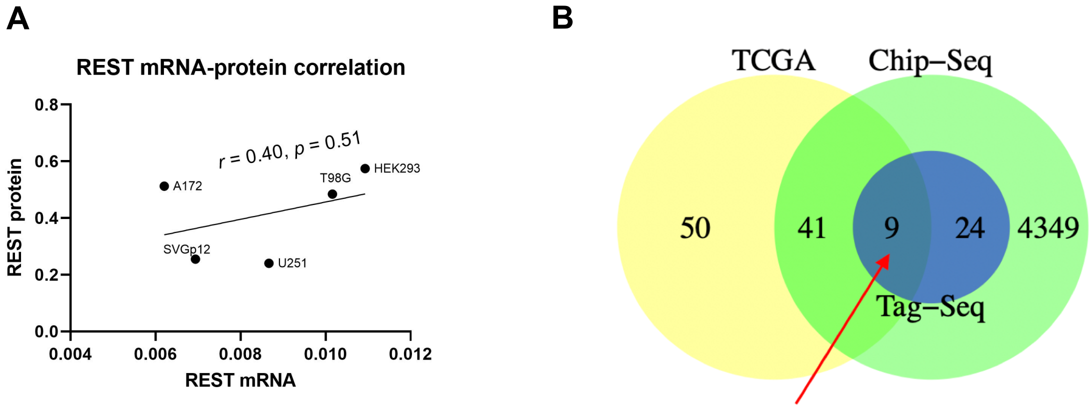

Figure S8. Assessment of functional level of REST in glioblastoma cells. **A,** Dot plot of mRNA and protein levels of REST across tested cell lines. Shown are mean values obtained from 3-4 independent biological replicates. Correlation was assessed using Pearson *r* coefficient, line of best fit represents linear regression line. *REST* gene expression was normalized by *ACTB* expression. REST protein level was assessed with Western blot and normalized by β-tubulin amount. **B**, The rationale for inclusion of specific genes in a REST-predictive gene panel (n = 9). Shown is overlap (indicated by red arrow) between three gene subsets: 1) top-100 anti-correlated with REST mRNA genes in TCGA-GBM dataset (yellow); 2) genes with REST binding site(s) in their genomic sequence based on analysis from Rockowitz et al 2015 [PMID 25990720] (green); 3) up-regulated genes in both REST-KO T98G and HEK293 (blue).

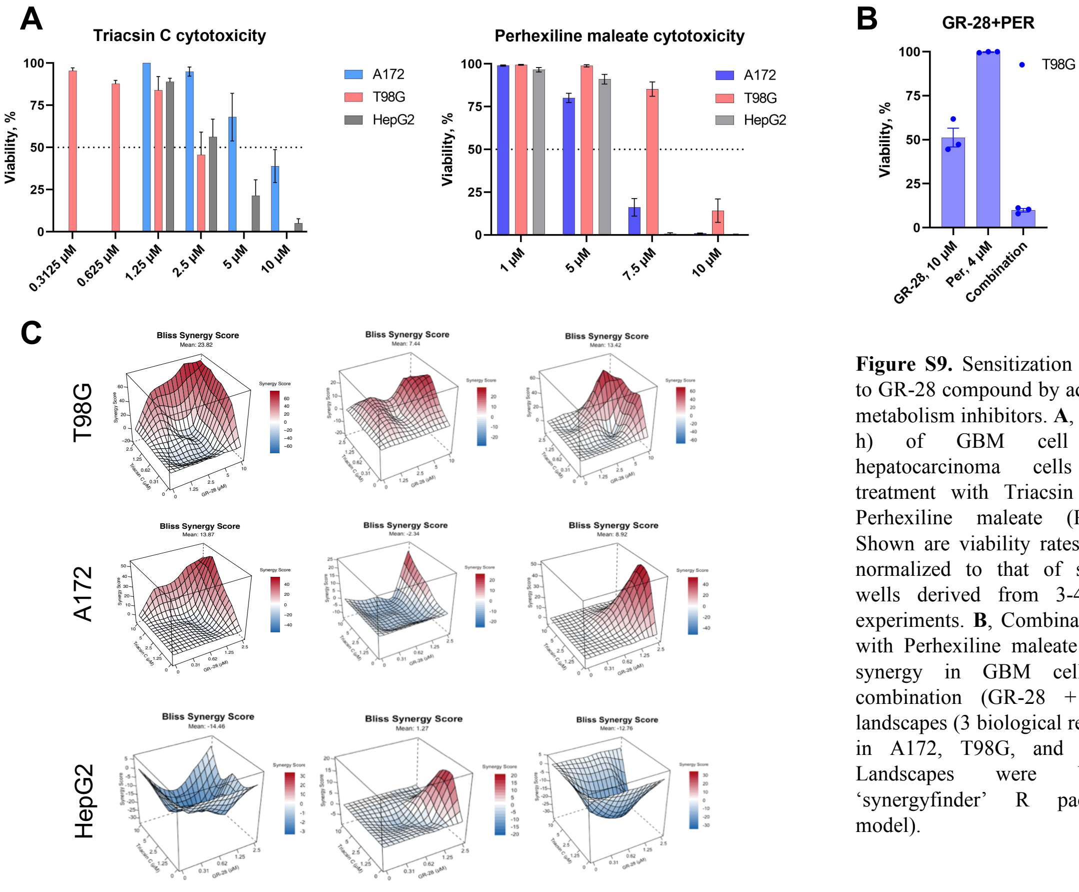

Figure S9. Sensitization of GBM cells to GR-28 compound by addition of lipid metabolism inhibitors. **A**, Sensitivity (72 h) of GBM cell lines and hepatocarcinoma cells to single treatment with Triacsin C (*left*) and Perhexiline maleate (PER) (*right*). Shown are viability rates (mean±SEM) normalized to that of solvent-control wells derived from 3-4 independent experiments. **B**, Combination of GR-28 with Perhexiline maleate (PER) shows synergy in GBM cells. **C**, Drug combination (GR-28 + Triacsin C) landscapes (3 biological replicates, 72 h) in A172, T98G, and HepG2 cells. Landscapes were built using 'synergyfinder' R package (Bliss model).

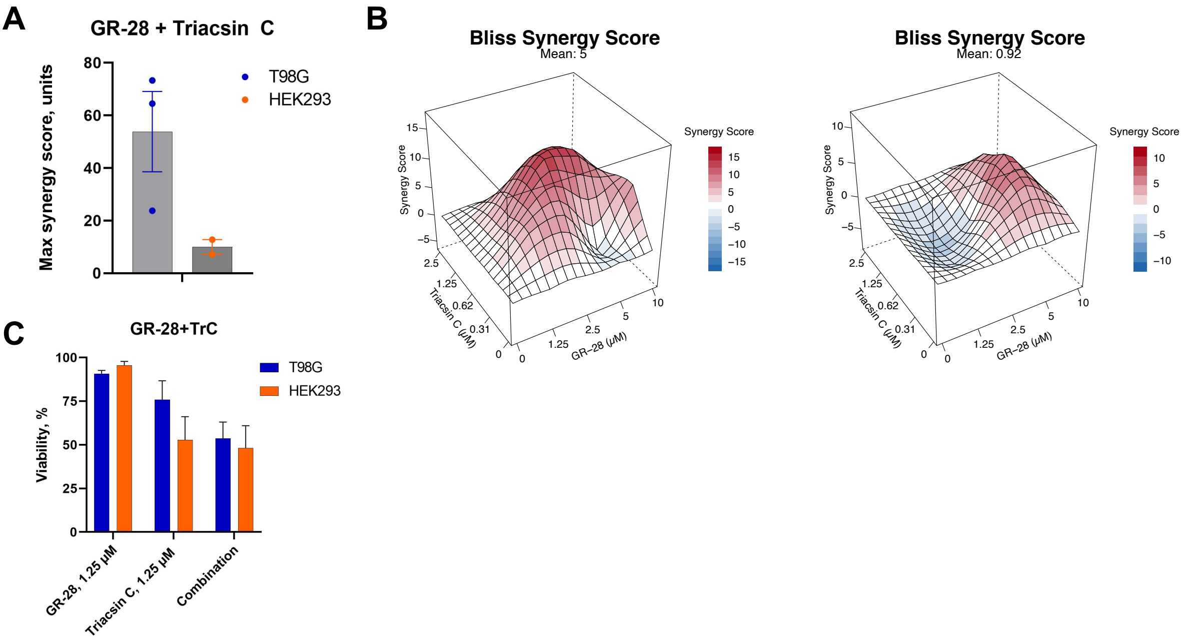

Figure S10. Sensitivity of non-cancerous HEK293 cells to a combination of GR-28+TrC.
A, Maximal synergy scores (mean±SEM) extracted from 2-3 independent drug combination landscapes (GR-28/Triacsin C) in T98G vs. HEK293 cells (72 h). **B**, Drug combination (GR-28 + Triacsin C) landscapes (2 biological replicates, 72 h) in HEK293 cells. Landscapes were built using 'synergyfinder' R package (Bliss model). **C**, Sensitivity of HEK293 cells to GR-28+TrC combination (72 h). Shown are viability rates (mean±SEM) normalized to that of solvent-control wells derived from 2 independent experiments. As HEK293 were somewhat more sensitive to Triacsin C, we could not achieve significant difference in survival vs. T98G.
