# Supplementary material for: Targeting of REST with rationally-designed small molecule compounds exhibits synergetic therapeutic potential in human glioblastoma cells
Source: BMC Biol. 2024 Apr 12;22:83. doi: 10.1186/s12915-024-01879-0 (PMC11015551; doi:10.1186/s12915-024-01879-0)
Supplement: Supplementary file 11 — Additional file 11. Original blots. [file 12915_2024_1879_MOESM11_ESM.pdf]

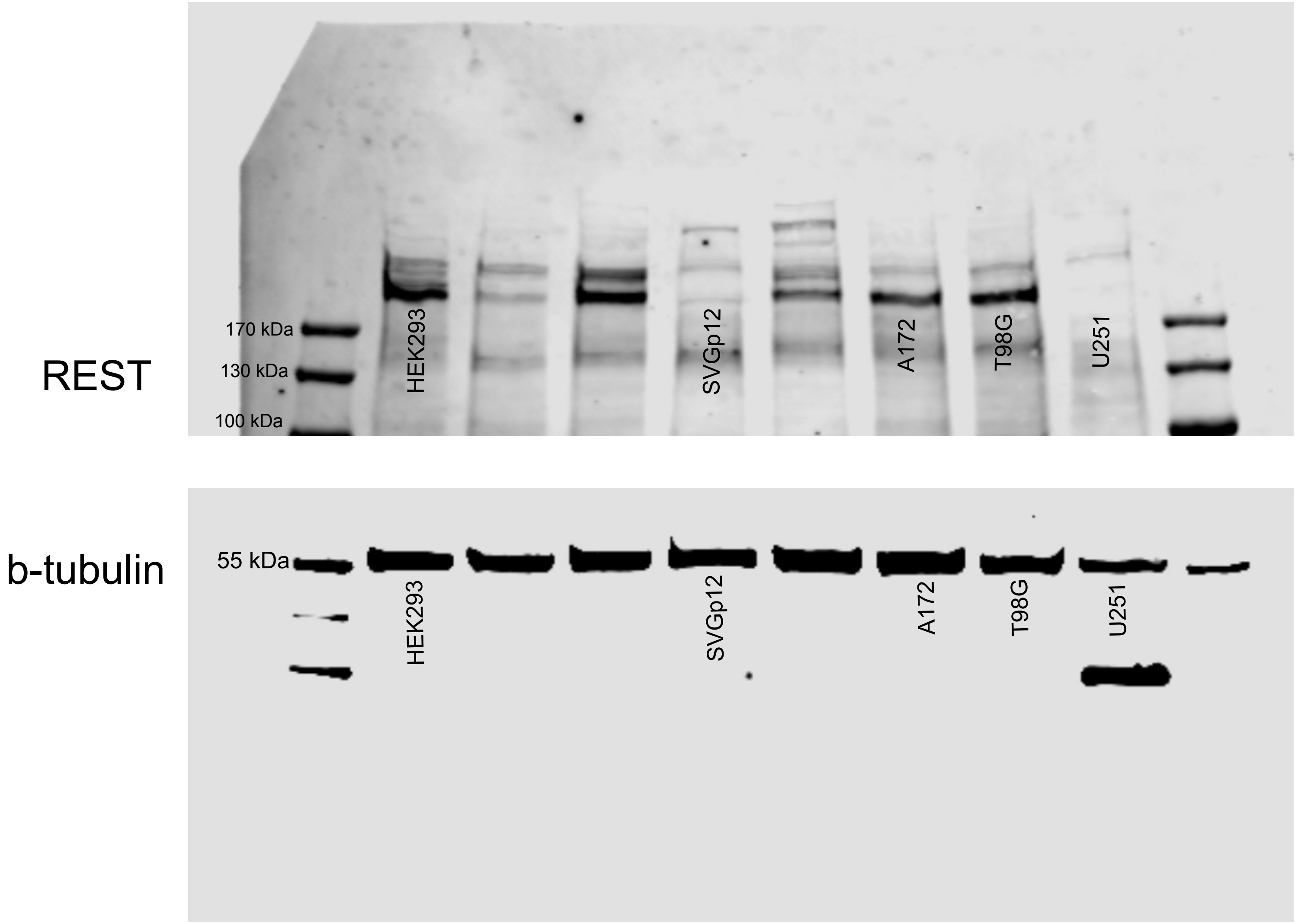
REST
170 kDa
130 kDa
100 kDa
HEK293
SVGp12
A172
T98G
U251
b-tubulin
55 kDa
HEK293
SVGp12
A172
T98G
U251

Figure 1C

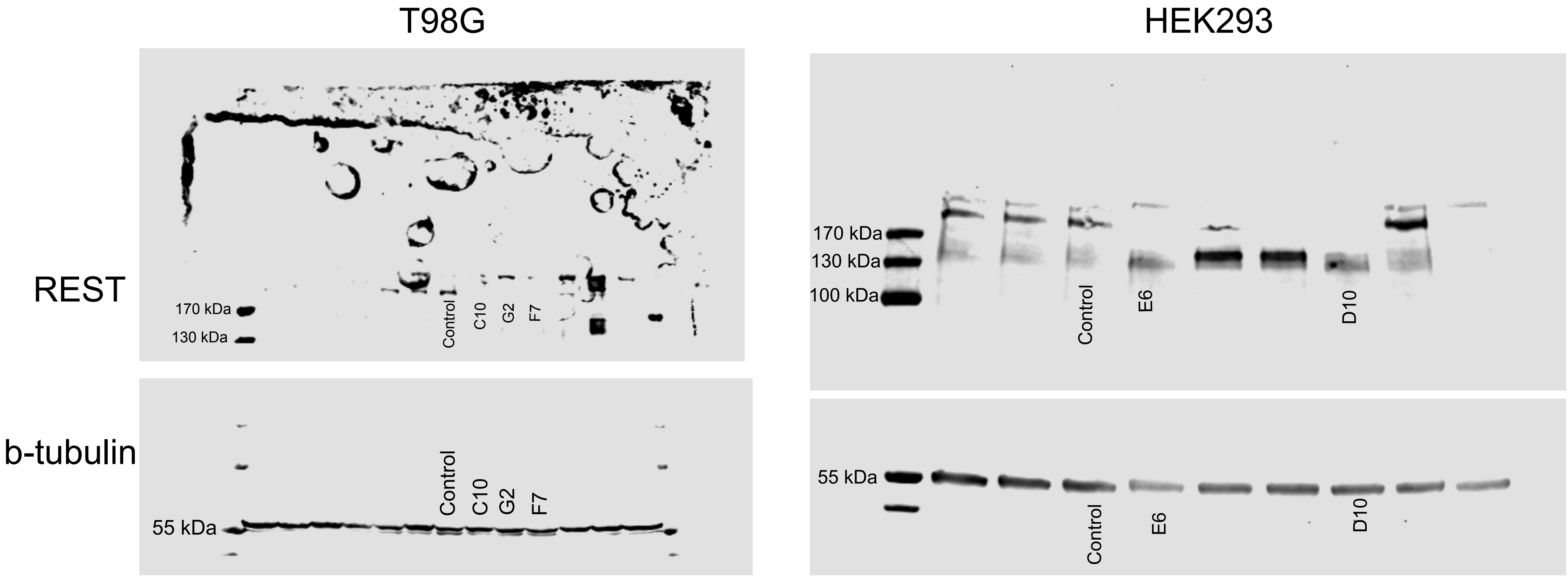
T98G
HEK293
REST
b-tubulin
170 kDa
130 kDa
100 kDa
55 kDa
Control
C10
G2
F7
E6
D10

Figure 1E

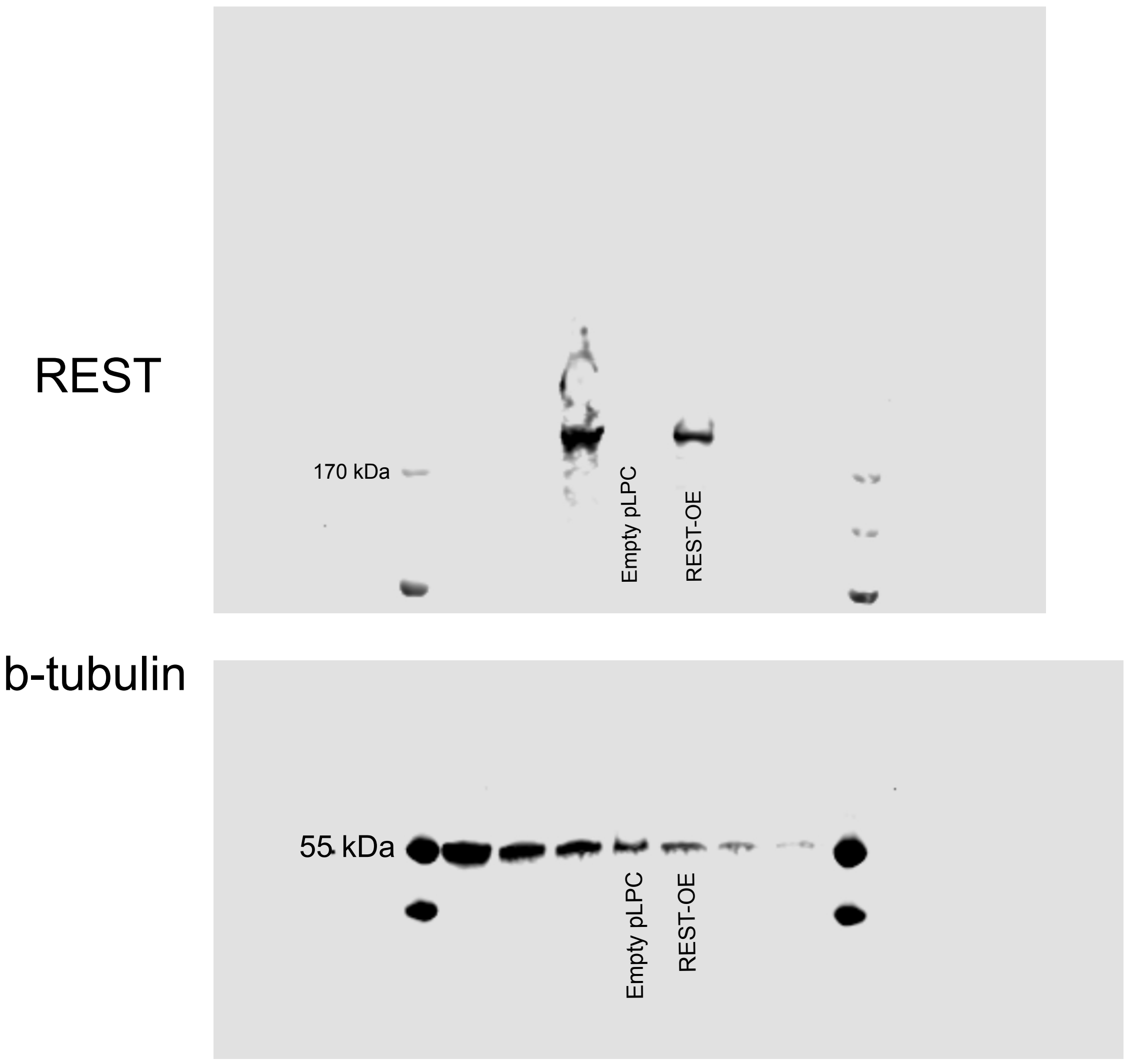
REST
170 kDa
Empty pLPC
REST-OE
b-tubulin
55 kDa
Empty pLPC
REST-OE

Figure 1G

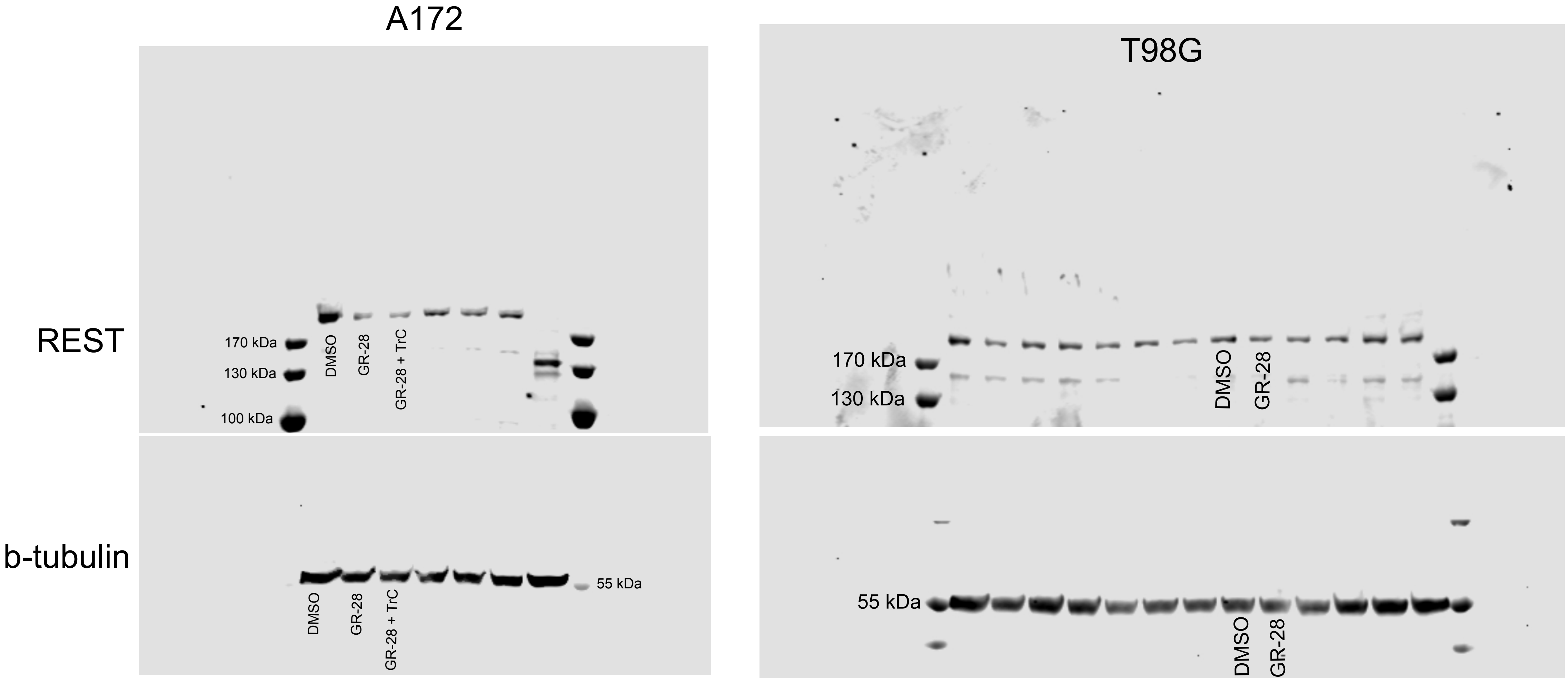
A172
T98G
REST
b-tubulin
170 kDa
130 kDa
100 kDa
DMSO
GR-28
GR-28 + TrC
55 kDa
170 kDa
130 kDa
DMSO
GR-28
55 kDa
DMSO
GR-28

Figure 4A

REST

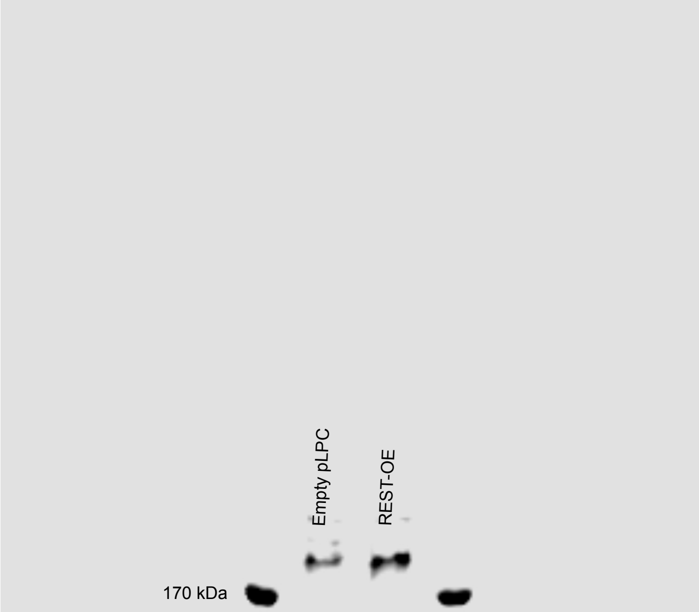

b-tubulin

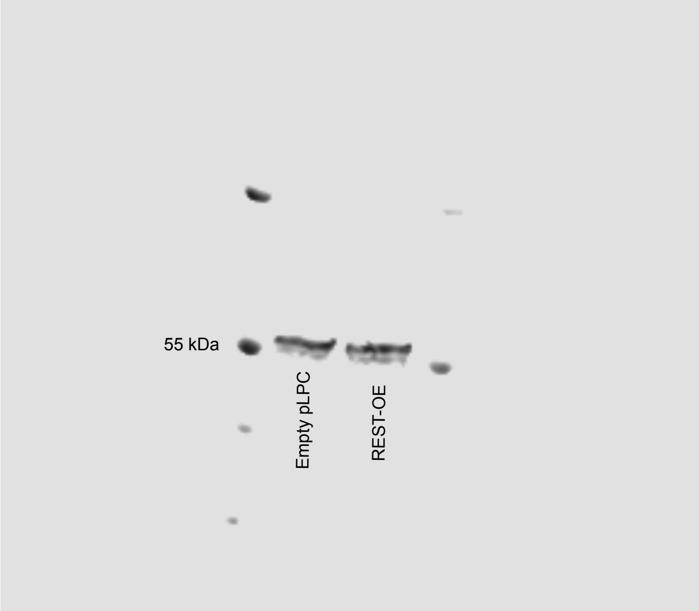

Figure 4E

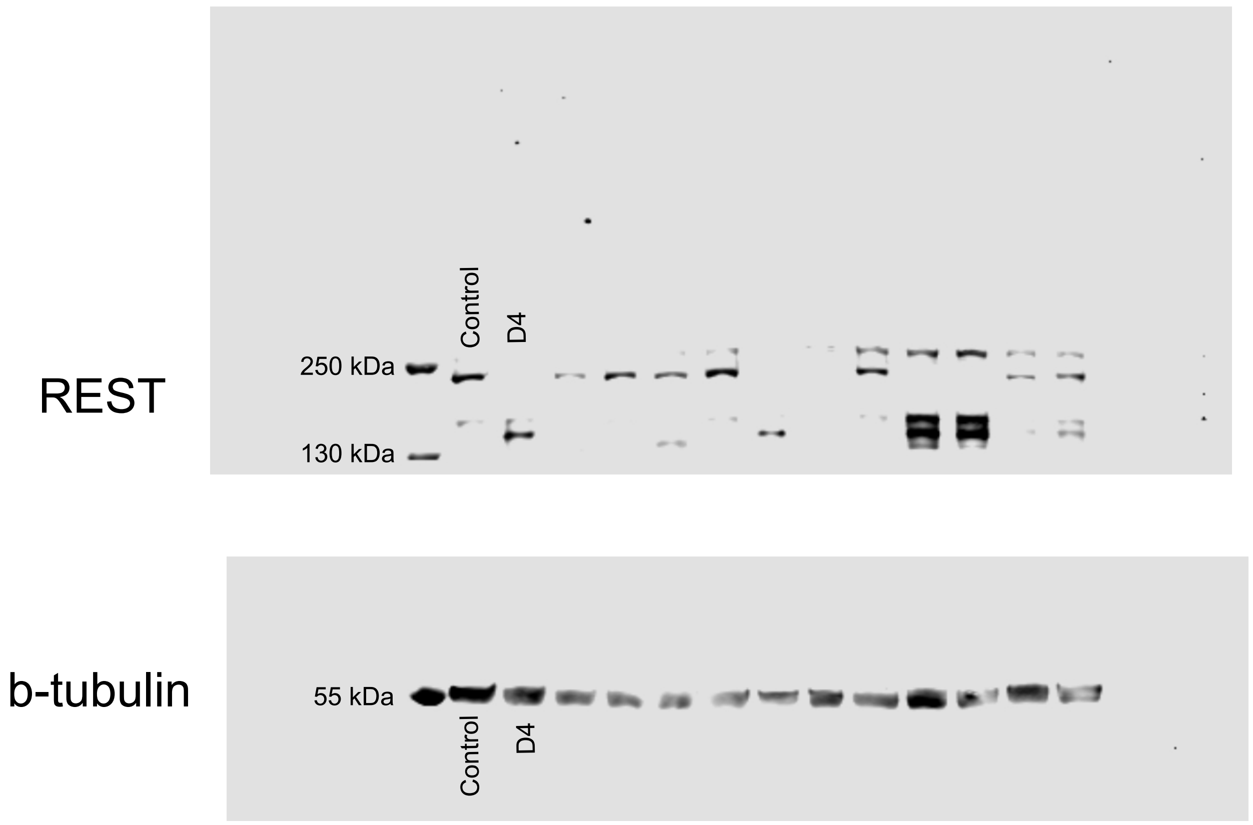

Figure 5A

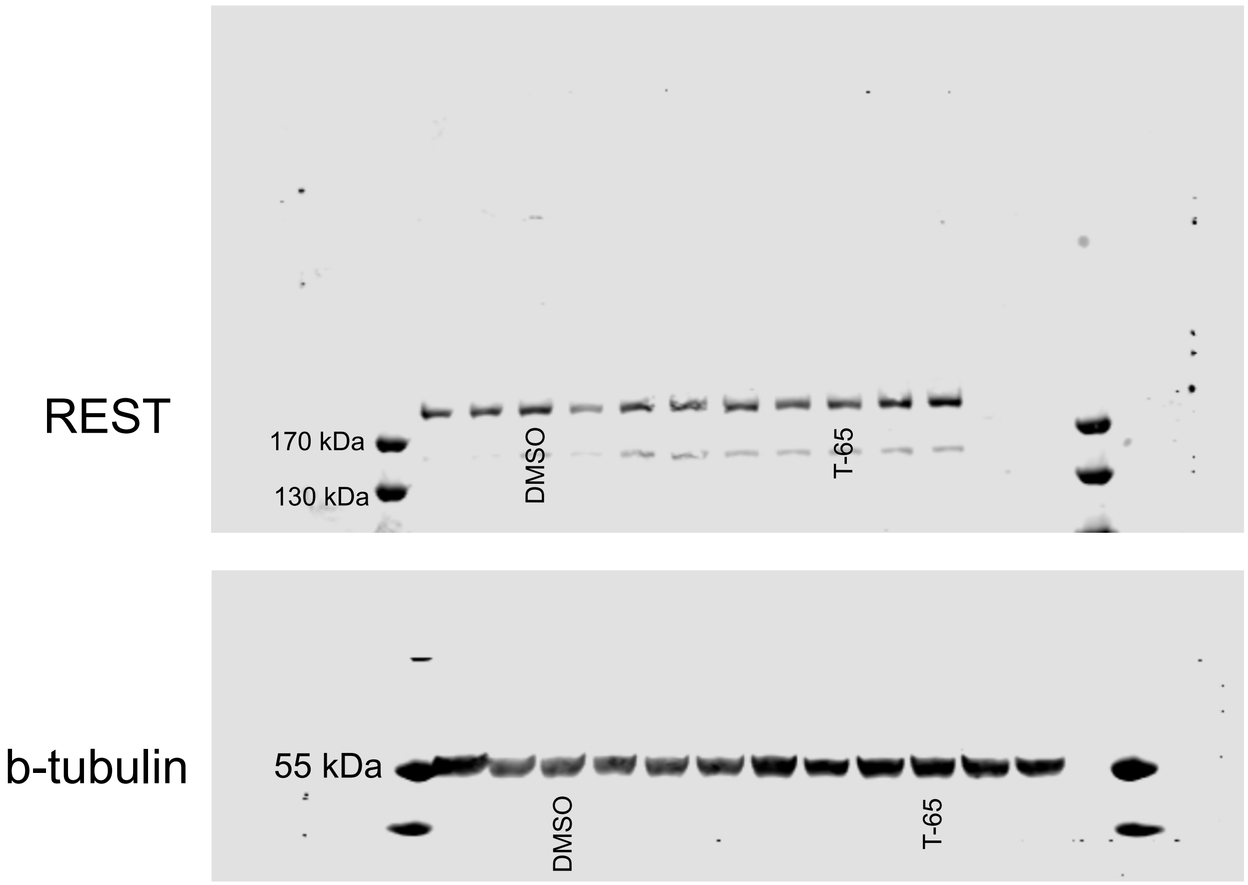

Figure S4D

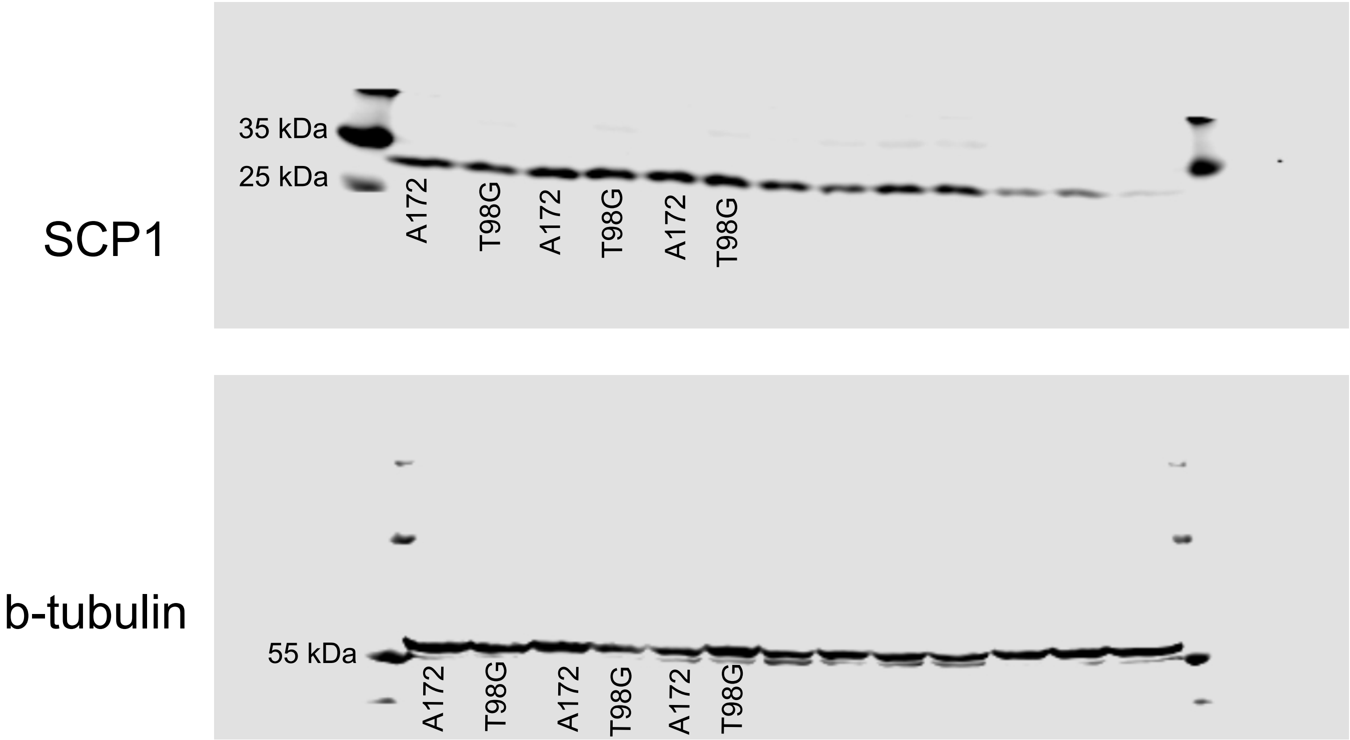
SCP1
35 kDa
25 kDa
A172
T98G
A172
T98G
A172
T98G
b-tubulin
55 kDa
A172
T98G
A172
T98G
A172
T98G

Figure S4E
